# MiR-129-5p exerts Wnt signaling-dependent tumor-suppressive functions in hepatocellular carcinoma by directly targeting hepatoma-derived growth factor *HDGF*

**DOI:** 10.1186/s12935-022-02582-2

**Published:** 2022-05-16

**Authors:** Nicole Huge, Thea Reinkens, Reena Buurman, Maria Sandbothe, Anke Bergmann, Hannah Wallaschek, Beate Vajen, Amelie Stalke, Melanie Decker, Marlies Eilers, Vera Schäffer, Oliver Dittrich-Breiholz, Engin Gürlevik, Florian Kühnel, Brigitte Schlegelberger, Thomas Illig, Britta Skawran

**Affiliations:** 1 Department of Human Genetics, Hannover Medical School, Carl-Neuberg-Straße 1, 30625 Hannover, Germany; 2 Research Core Unit Genomics, Hannover Medical School, Hannover, Germany; 3 Department of Gastroenterology, Hepatology and Endocrinology, Hannover Medical School, Hannover, Germany; 4 Hannover Unified Biobank (HUB), Hannover Medical School, Hannover, Germany

**Keywords:** HCC, HDAC inhibitors, miRNA sequencing, ERK signaling, Wnt signaling, miRNA replacement therapy, Personalized therapy

## Abstract

**Background:**

In hepatocellular carcinoma (HCC), histone deacetylases (HDACs) are frequently overexpressed. This results in chromatin compaction and silencing of tumor-relevant genes and microRNAs. Modulation of microRNA expression is a potential treatment option for HCC. Therefore, we aimed to characterize the epigenetically regulated miR-129-5p regarding its functional effects and target genes to understand its relevance for HCC tumorigenesis.

**Methods:**

Global miRNA expression of HCC cell lines (HLE, HLF, Huh7, HepG2, Hep3B) and normal liver cell lines (THLE-2, THLE-3) was analyzed after HDAC inhibition by miRNA sequencing. An in vivo xenograft mouse model and in vitro assays were used to investigate tumor-relevant functional effects following miR-129-5p transfection of HCC cells. To validate hepatoma-derived growth factor (*HDGF*) as a direct target gene of miR-129-5p, luciferase reporter assays were performed. Survival data and *HDGF* expression were analyzed in public HCC datasets. After siRNA-mediated knockdown of *HDGF*, its cancer-related functions were examined.

**Results:**

HDAC inhibition induced the expression of miR-129-5p. Transfection of miR-129-5p increased the apoptosis of HCC cells, decreased proliferation, migration and ERK signaling in vitro and inhibited tumor growth in vivo*.* Direct binding of miR-129-5p to the 3′UTR of *HDGF* via a noncanonical binding site was validated by luciferase reporter assays. *HDGF* knockdown reduced cell viability and migration and increased apoptosis in Wnt-inactive HCC cells. These in vitro results were in line with the analysis of public HCC datasets showing that *HDGF* overexpression correlated with a worse survival prognosis, primarily in Wnt-inactive HCCs.

**Conclusions:**

This study provides detailed insights into the regulatory network of the tumor-suppressive, epigenetically regulated miR-129-5p in HCC. Our results reveal for the first time that the therapeutic application of mir-129-5p may have significant implications for the personalized treatment of patients with Wnt-inactive, advanced HCC by directly regulating *HDGF*. Therefore, miR-129-5p is a promising candidate for a microRNA replacement therapy to prevent HCC progression and tumor metastasis.

**Supplementary Information:**

The online version contains supplementary material available at 10.1186/s12935-022-02582-2.

## Background

Hepatocellular carcinoma (HCC) is the most common type of primary liver cancer and the fourth leading cause of cancer-related death worldwide [1]. In the majority of cases, HCC develops from chronic inflammation in a cirrhotic liver and is associated with etiological factors such as viral hepatitis B and C infections, chronic alcohol consumption or ingestion of aflatoxins [2]. Unfortunately, most patients are diagnosed at an advanced stage with very limited treatment options. Until recently, multi-kinase inhibitors such as sorafenib and lenvatinib have been the standard of care for these patients [3, 4]. Since May 2020, the combination of atezolizumab (immunotherapy) plus bevacizumab (anti-VEGF) has become the new reference standard in the first-line treatment of HCC [3, 5]. However, strong side effects, low response rates and drug resistance pose additional challenges for HCC therapy [6]. Therefore, the development of new targeted therapeutic strategies is an important goal of HCC research.

Several studies have reported significant upregulation of histone deacetylases (HDACs) in HCC [7–9] suggesting that these epigenetic modifiers may provide an attractive option for HCC therapy. HDACs remove acetyl groups on N-terminal lysine residues of histones leading to a highly condensed chromatin and transcriptional silencing of the respective genomic regions. Notably, the loss of acetylation at Lys16 of histone H4 is one of the hallmarks of human cancers, including HCC, which implicates a critical role of HDAC activity in tumorigenesis [10]. HDAC inhibitors (HDACi), such as Vorinostat (SAHA) and Romidepsin (FK228), have the potential to inhibit tumor growth and metastasis as well as to induce apoptosis and cell cycle arrest by disrupting multiple signaling pathways [11]. Four HDACi have been approved for treatment of hematologic malignancies by the US Food and Drug Administration (FDA) and several HDACi are subjects of clinical trials for solid tumors including HCC [12]. However, knowledge of the affected signaling pathways and effects on the expression of cancer-relevant genes and microRNAs (miRNAs) is still incomplete and requires further investigation.

HDACi have previously been reported to alter the expression of miRNAs in a number of cancers [13]. MicroRNAs are small non-coding RNAs that act as endogenous gene silencers by complementarily binding to the 3′ untranslated region (3′UTR) of mRNAs. As part of the RNA-induced silencing complex (RISC), miRNAs either repress the translation or promote the degradation of their target mRNAs. In the context of cancer, miRNAs may serve as diagnostic biomarkers and even therapeutic tools because of their critical regulatory function in a variety of tumor-relevant pathways such as proliferation, apoptosis, differentiation and migration [14]. Therefore, miRNA-based cancer therapy is a promising treatment option that may improve patient outcomes.

Previously, we reported that the expression profiles of miRNAs in HCC cell lines are regulated by epigenetic mechanisms and have shown that the tumor-suppressive effects of HDACi in HCC are partly caused by the re-expression of the miR-449 family [8, 15]. We have identified miR-129-5p as strongly upregulated following HDAC inhibition in HCC cell lines. This miRNA is encoded by two genes, *miR-129-1* and *miR-129-2* located near a fragile site on chromosome 7 [16] and in a CpG island on chromosome 11 [17], respectively. Interestingly, reduced miR-129-5p expression caused by *miR-129-2* promoter hypermethylation is often observed in solid tumors such as HCC [18, 19], gastric [20], breast [21] and colorectal cancer [22]. An already identified target gene of miR-129-5p is the transcription factor *SOX4*, which is involved in the regulation of TGF-β-mediated epithelial to mesenchymal transition (EMT) [15, 23]. EMT evokes a change from a polarized epithelial phenotype, in which cells express epithelial markers including E-cadherin (*CDH1*), to a mesenchymal state in which cell–cell contact is lost and mesenchymal marker Vimentin (*VIM*) is expressed [23]. TGF-β is a potent inducer of EMT in a variety of human cancers and mediates the induction of a mesenchymal phenotype during EMT that is controlled by transcriptional activator *SOX4* [23, 24]. However, the miR-129-5p regulatory network is not yet fully understood. The characterization of target genes is crucial to elucidate the regulatory network of a miRNA. Putative target genes may be identified by global expression analysis after miRNA transfection but need to be validated by confirming a direct interaction of miRNA and target mRNA. This may be accomplished by luciferase assays or AGO2-IP, providing evidence for a direct binding of the miRNA to its target mRNA in the RISC [25].

Here, we characterize the epigenetically deregulated miR-129-5p and analyze its functional effects and target genes in hepatocellular carcinoma.

## Methods

### Cell culture and transfection

Liver cancer cell lines, HLE, HLF, Huh7 and Huh6 were kindly provided by Professor Nam-Ho Huh (Okayama University, Okayama, Japan). The HCC cell line HepG2 and normal liver cell lines, THLE-2 and THLE-3 were purchased form ATCC (Manassas, VA, USA). The HCC cell line Hep3B was kindly provided by Professor Dr. Florian Kühnel (Hannover Medical School, Hannover, Germany). All cell lines were cultured at 37 °C and 5% CO_2_ in a humidified incubator. Liver cancer cell lines HLE, HLF, Huh7, HepG2, Hep3B and Huh6 were cultured in Dulbecco’s Modified Eagle Medium with 10% FCS, 2 mM l-glutamine, and 100 U/mL penicillin/streptomycin. For characterization of HCC cell lines refer to Nwosu et al*.* [26]. Normal liver cell lines (THLE-2, THLE-3) were cultured in Bronchial Epithelial Cell Growth Basal Medium (Lonza, Basel, Switzerland) with 70 ng/mL phosphoethanolamine, 5 ng/mL epidermal growth factor, and 10% FCS.

For HDAC inhibition, cells were treated with 2 µM suberoyl anilide hydroxamic acid (SAHA), 35 nM romidepsin (FK228) or 100% ethanol vehicle control based on experiments of Yang et al*.* [27] and Furumai et al*.* [28]. For analysis of TGF-β-induced *SOX4* expression, cells were treated with 5 ng/mL TGF-β or 0.1% BSA vehicle control [15].

Cells were transfected with 10 nM siRNAs against *HDGF* or 50 nM miR-129-5p mimics (Qiagen, Hilden, Germany) using HiPerFect Transfection Reagent (Qiagen). As non-targeting control, AllStars Negative Control (Qiagen) was used, hereinafter referred to as miR-control or si-control. Medium was renewed 24 h after transfection. All cell lines were authenticated using STR profiling and all experiments were performed with mycoplasma-free cells.

### MicroRNA sequencing

**Fig. 1:**
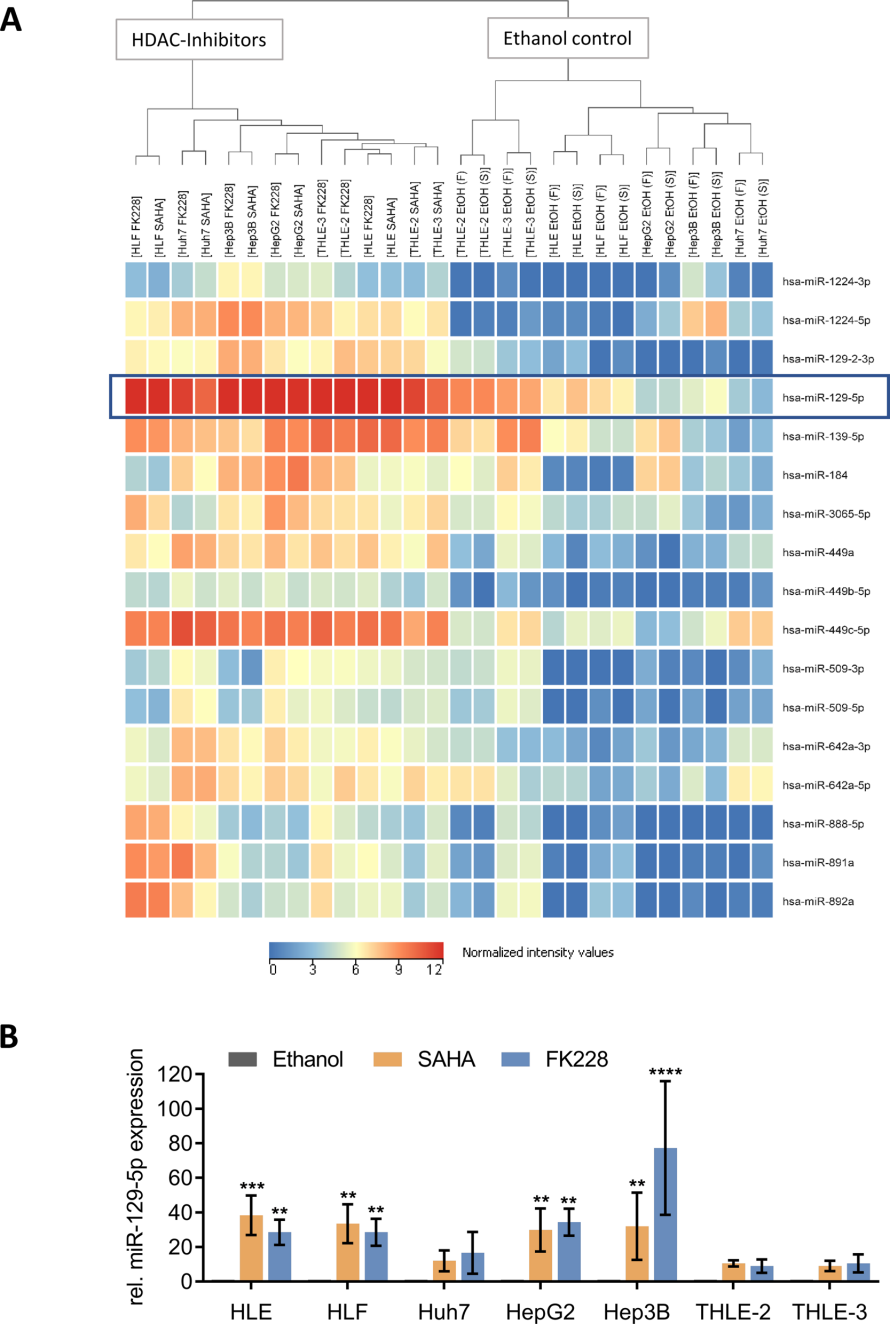
Expression of miR-129-5p is regulated by histone acetylation. **A** Expression of microRNAs after HDAC inhibition was determined by microRNA sequencing. HCC and normal liver cell lines were treated with 2 µM SAHA, 35 nM FK228 or ethanol vehicle control for 24 h. Seventeen miRNAs were upregulated (FC ≥ 2) after SAHA and FK228 treatment in all examined HCC cell lines and were hierarchically clustered. **B** Induction of miR-129-5p expression after HDAC inhibition was validated by quantitative Real-Time PCR. Results were normalized to ethanol controls and values of ethanol-treated control cells were set to 1 to enable a better comparability of the data. **p < 0.01, ***p < 0.001, ****p < 0.0001; two-way ANOVA with Dunnett’s multiple comparisons test

Sequencing libraries were generated with the NEBNext® Multiplex Small RNA Library Prep Kit for Illumina (New England Biolabs, Ipswich, USA). Enrichment and size distribution of the libraries were quality-assessed using Bioanalyzer DNA 1000 Assay (Agilent, Santa Clara, USA). Single-read sequencing was performed on an Illumina NextSeq 550 sequencer using a High Output Flowcell for 75 bp single reads (Illumina, San Diego, USA). BCL files were converted to FASTQ files using bcl2fastq Conversion Software version 2.17.1.14 (Illumina). The FASTQ files were adapter- and quality-trimmed using Trim Galore! (version 0.4.1) with default settings as described in the User Guide except for the setting of the quality cutoff (-q/–quality), which was set to a Phred score of 15 and the length cutoff (–length), which was set to 6 bp. Trim Galore! used Cutadapt (version 1.9.1) as subroutine. Quality control of FASTQ files was performed by FastQC (version 0.11.4) before and after trimming. Final data analysis and visualization were performed with StrandNGS (version 3.0.1). Values of biological replicates were averaged and differentially expressed miRNAs were identified by fold change analysis (FC ≥ 2). The miRNAs depicted in Fig. 1A were upregulated after both HDACi treatments (SAHA and FK228) in all five tested HCC cell lines (HLE, HLF, Huh7, HepG2, Hep3B).

### Global histone acetylation, cell viability, apoptosis and migration assay

Global histone acetylation was determined by the Cyclex Cellular Histone Acetylation Kit (Cyclex Co, Nagano, Japan) according to the manufacturer’s instructions. Cell viability and apoptosis were measured in triplicate every 24 h using WST-1 Proliferation Reagent (Roche, Basel, Switzerland) and the Caspase3/7 Glo Assay (Promega, Madison, WI, USA), respectively. Cell migration was determined using transwell assays (8 µm pore size, Corning, NY, USA) as has been described previously [15].

### Xenograft mouse model

Huh7 cells were harvested 72 h after transient transfection with 50 nM miRNA mimics. Subsequently, 5 × 10^6^ cells were injected subcutaneously into the left flank of 6-week-old Naval Medical Research Institute (NMRI)^nu/nu^ mice (n = 7 for each condition). Tumor volume (V) was measured and was calculated with the formula V = (length × width^2^) × 0.5. Animal experiments were conducted according to the legal guidelines of animal experimentation and care (TierSchG) in Germany and were approved by the responsible institutional board of the MHH in coordination with the competent supervisory authority (LAVES).

### Analysis of mRNA, miRNA and protein expression

Global mRNA expression profiling by microarray was performed as previously described [15].

To quantify mRNA and miRNA expression levels by quantitative real-time PCR (qRT-PCR), total RNA was isolated with the Direct-zol RNA MiniPrep Kit (Zymo Research, Irvine, CA, USA). RNA was transcribed into cDNA using the High-Capacity cDNA Reverse Transcription Kit (Thermo Fisher Scientific, Waltham, MA, USA). Relative mRNA and miRNA expression was measured in triplicate using TaqMan Gene Expression and MicroRNA Assays (Life Technologies, Carlsbad, CA, USA). For normalization, *TBP* and *RNU6B* were used as reference genes for mRNA and miRNA expression analyses, respectively.

For protein analysis, whole cell lysates were prepared with RIPA buffer using equal cell numbers per sample. Protein levels were analyzed using a standard western blot protocol with antibodies against HDGF (AF1606; R&D Systems, Minneapolis, MN, USA), SOX4 (C15310129; Diagenode, Seraing, Belgium), ERK 1/2 (4695; Cell Signaling Technology), phospho-ERK 1/2 (4370; Cell Signaling Technology), cofilin (5175; Cell Signaling Technology), β-actin (3700; Cell Signaling Technology), and α-actinin (4233; Cell Signaling Technology).

### Luciferase reporter assay

Potential miR-129-5p binding sites within the *HDGF* 3′UTR were identified by TargetScan [29] or IntaRNA [30]. Luciferase reporter vectors were constructed containing the 3ʹUTR of *HDGF* either with intact miR-129-5p binding sites (*HDGF*-3′UTR) or with mutated miR-129-5p binding sites (*HDGF*-TargetScan-mut or *HDGF*-IntaRNA-mut). For this, a 1185 bp [*HDGF*-3′UTR (68–1253)] and a 488 bp fragment [*HDGF*-3′UTR (765–1253)] containing all three potential miR-129-5p binding sites were PCR-amplified from cDNA of HLE cells. Three 282 bp fragments [*HDGF*-3′UTR, *HDGF*-IntaRNA1-mut, *HDGF*-IntaRNA2-mut (971–1253)] containing wildtype IntaRNA sites, mutated IntaRNA binding site 1 and mutated IntaRNA binding site 2, respectively, were purchased as gBlocks Gene Fragments (Integrated DNA Technologies, Coralville, IA, USA). All fragments were digested with XbaI and FseI, and cloned downstream of firefly luciferase into the pGL3-Promoter vector (Promega, Madison, WI, USA). The binding site predicted by TargetScan was mutated using the QuikChange Site-Directed Mutagenesis Kit (Agilent). For transfection, 8000 HEK293 cells were seeded in white 96-well plates. The next day, combinations of 20 nM miRNA mimics (miR-control, miR-129-5p) and 25 ng luciferase reporter vectors (pGL3-Promoter, *HDGF*-3′UTR_68–1253_, *HDGF*-3′UTR_765–1253_, *HDGF*-3′UTR_971–1253_, *HDGF*-TargetScan-mut, *HDGF*-IntaRNA1-mut, or *HDGF*-IntaRNA2-mut) were transfected in triplicate using Lipofectamine 2000 (Life Technologies). For normalization, pGL4.70 (Promega) with an EF1α promoter inserted at the XhoI restriction site upstream of renilla luciferase was co-transfected in all conditions. Luciferase activities were measured with the DualGlo Luciferase Assay System (Promega) 24 h after transfection.

### AGO2 immunoprecipitation (AGO2-IP)

MiR-129-5p versus control transfected HLE cells were lysed in 3× NP40 lysis buffer, incubated for 20 min on ice and centrifuged (15 min, 13,000×*g* and 4 °C). For the input, 20 µL of the supernatant was stored. Dynabeads Protein G (Thermo Fisher Scientific, Braunschweig, Germany) were prepared by washing three times with citrate phosphate buffer and 1 h incubation at 4 °C with control IgG or AGO2 antibody. The lysates were incubated with dynabeads for 1 h at 4 °C. Then, lysates were washed three times with IP wash buffer and high salt wash buffer and two times with phosphatase wash buffer and PNK buffer. After successful IP, RNA was isolated and subjected to qRT-PCR for validation.

### Analysis of public datasets

To analyze the expression of *HDGF* in HCC and adjacent liver tissue, expression levels of the NCBI GEO data sets GSE22058, GSE25097, and GSE54236 were downloaded (https://www.ncbi.nlm.nih.gov/geo/). In addition, log2-transformed, normalized mRNA expression values (RSEM, Illumina HiSeq_RNASeqV2) and clinical data of the TCGA-LIHC cohort were downloaded from the Cell Index Database CELLX [31] and the GDC portal (https://portal.gdc.cancer.gov/), respectively. Survival data of the TCGA-LIHC cohort along with *HDGF* expression levels were obtained from http://www.oncolnc.org/ [32] and the expression was categorized into high (upper median) or low (lower median). Furthermore, the TCGA-LIHC cohort was stratified into cases without or with Wnt/β-catenin signaling activation according to Sanchez-Vega et al*.* [33].

### Statistics

Data are represented as mean ± standard deviation (SD) of at least three independent experiments unless stated otherwise. Statistical significance was determined with GraphPad Prism (GraphPad Software, Version 9) by two-tailed Student’s *t* tests or by one-way or two-way ANOVA with Dunnett’s multiple comparisons test or Tukey’s multiple comparison test as indicated. For xenograft mouse experiments statistical significance was determined by two-way ANOVA with Šídák’s multiple comparisons test.

## Results

### Expression of miR-129-5p is regulated by histone acetylation

We have previously reported that miR-129-5p is one of the strongest upregulated miRNAs following TSA-mediated HDAC inhibition in HCC cell lines [8]. Since TSA is only used in laboratory experiments because of its toxicity [34], we aimed to analyze miRNA expression of liver cell lines after HDAC inhibition by SAHA and FK228, two HDACi approved by the FDA for the treatment of cutaneous T-cell lymphoma. We identified several miRNAs that were induced upon SAHA and FK228 treatment through miRNA sequencing (Fig. 1). Among them, miR-129-5p was the most highly upregulated miRNA, which was primarily attributable to an upregulation of *pri-miR-129-2* expression after SAHA or FK228 treatment (Additional file 1: Fig. S1). The upregulation of miR-129-5p expression after HDAC inhibition was validated by qRT-PCR in all analyzed HCC cell lines (Fig. 1B). Similar to TSA [8], SAHA and FK228 increased acetylation and apoptosis while decreasing cell viability of HCC cells (Additional file 1: Fig. S2). Our results demonstrate that miR-129-5p expression is epigenetically regulated by histone acetylation.

### miR-129-5p exerts distinct tumor-suppressive functions in vitro and in vivo

**Fig. 2:**
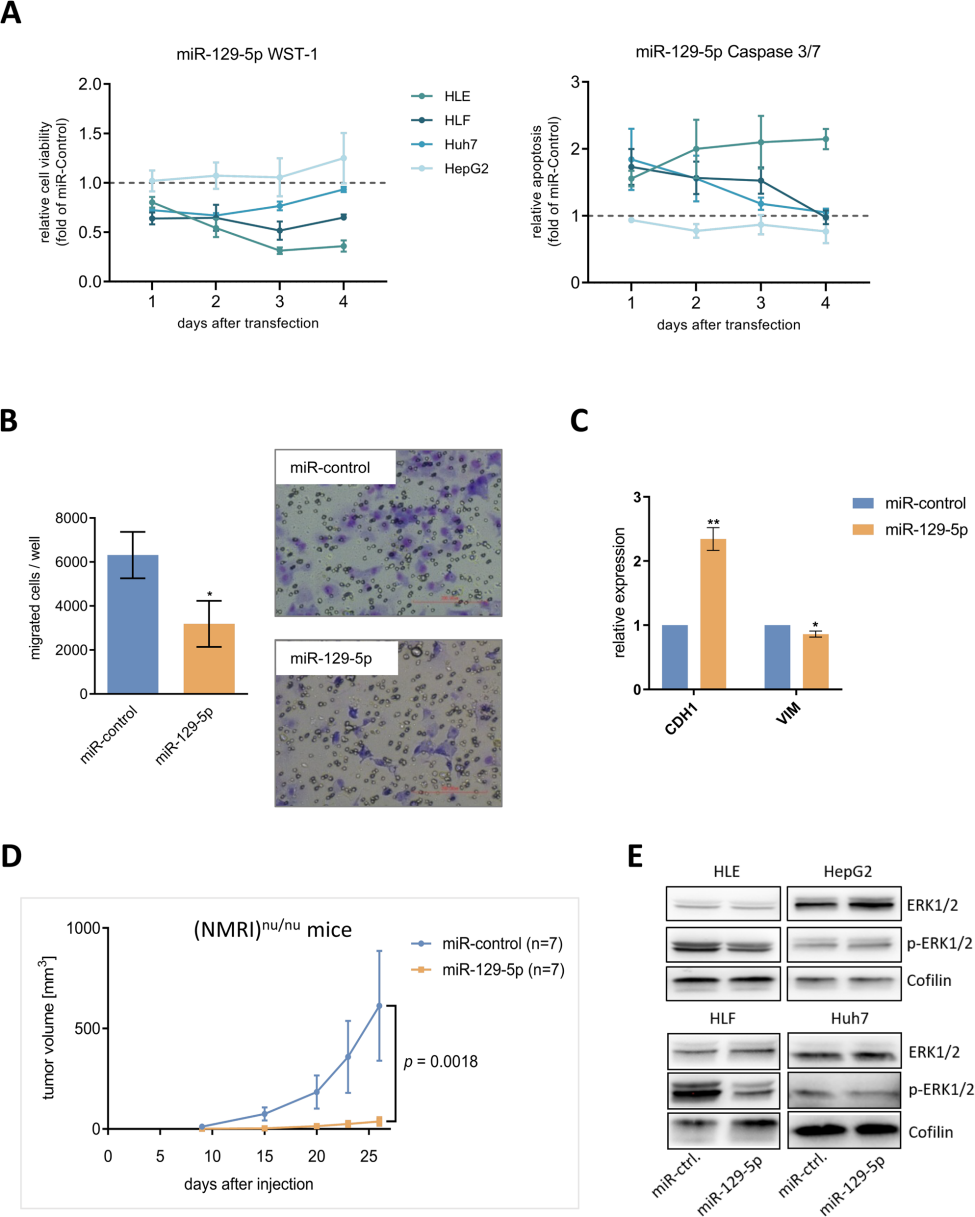
miR-129-5p exerts distinct tumor-suppressive functions in vitro and in vivo. **A** HLE, HLF, Huh7, and HepG2 cells were transfected with 50 nM miR-129-5p mimics. Cell viability was analyzed by WST-1 assay and normalized to miR-control (dotted line). Apoptosis was analyzed by Caspase 3/7 assay and normalized to cell viability and miR-control (dotted line). Data are represented as mean ± SEM. **B**, **C** HLE cells were transfected with 50 nM miR-129-5p mimics or miR-control. 48 h after transfection, cell migration (**B**) was analyzed by transwell assay and expression of *CDH1* and *VIM* (**C**) was measured by qRT-PCR using the ΔΔCT method. *p < 0.05, **p < 0.01; two-tailed Student’s t test; Scale bar = 200.00 µm. **D** Huh7 cells were transfected with 50 nM miRNA-129-5p mimics. After 72 h, cells were injected into the flanks of NMRI^nu/nu^ mice and tumor growth was monitored (n = 7 for each group). Data are represented as mean ± SEM; two-way ANOVA with Šídák’s multiple comparisons test. **E** HLE, HLF, Huh7, and HepG2 cells were transfected with miR-129-5p mimic. 48 h after transfection, protein expression of p-ERK1/2 and ERK1/2 was analyzed by western blotting with cofilin as loading control; Gels were processed in parallel. Densitometric analysis of western blot assays is shown in Additional file 1: Fig. S9A

To analyze tumor-relevant functional effects of miR-129-5p, we transiently transfected HCC cell lines HLE, HLF, Huh7 and HepG2 with miR-129-5p mimics. Cell viability was significantly reduced in HLE, HLF and Huh7 cells (Fig. 2A). Apoptosis was increased in HLE cells and, albeit less pronounced, also in HLF and Huh7 cells (Fig. 2A). Cell viability and apoptosis were not affected by miR-129-5p transfection in Wnt-active HepG2 cells. This demonstrates that miR-129-5p acts more specifically than HDAC inhibitors that cause an increase of apoptosis in HepG2 cells (Additional file 1: Fig. S2B, C) due to their global effect on chromatin and non-histone protein substrates, leading to a deregulation of a variety of miRNAs and genes [35]. Moreover, we observed that miR-129-5p reduced HLE cell migration capacity by 50% (Fig. 2B) and induced the expression of the epithelial marker *CDH1* while slightly decreasing the expression of the mesenchymal marker *VIM* (Fig. 2C). Notably, the in vitro results were confirmed in vivo using a xenograft mouse model. MiR-129-5p strongly reduced tumor growth in (NMRI)^nu/nu^ mice (Fig. 2D).

Subsequently, we aimed to further elucidate the downstream mechanisms of miR-129-5p causing the inhibition of HCC cell migration. Therefore, we analyzed the influence of miR-129-5p on *SOX4* expression and TGF-β-mediated cell migration. The transcription factor *SOX4* is an already identified target gene of miR-129-5p that is involved in the regulation of TGF-β-mediated epithelial to mesenchymal transition (EMT) and migration [23, 24]. Transfection of miR-129-5p downregulated *SOX4* expression (Additional file 1: Fig. S3A, B) and abrogated the TGF-β-mediated *SOX4* induction (Additional file 1: Fig. S3C) in HLE and HLF cells. In addition, miR-129-5p transfection reduced the basal migration of HLE and HLF cells and, more importantly, decreased the TGF-β-mediated cell migration (Additional file 1: Fig. S3D). Therefore, we conclude that miR-129-5p inhibits the TGF-β-mediated cell migration by downregulating *SOX4* in the TGF-β pathway.

Zeng et al*.* have implicated the involvement of miR-129-5p in the regulation of ERK signaling [36]. Consequently, we evaluated the effects of miR-129-5p on ERK activation in HLE, HLF, Huh7, and HepG2 cells by analyzing the expression levels of total and phosphorylated ERK1/2 (p-ERK) using western blots (Fig. 2E). In its phosphorylated form, ERK1/2 enhances growth and survival of HCC [37]. The protein expression level of p-ERK1/2 was decreased in HLE, HLF, and Huh7 cells after miR-129-5p transfection, while no reduction of total ERK1/2 was observed. In HepG2 cells, levels of total and phosphorylated ERK1/2 were slightly augmented after miR-129-5p transfection (Fig. 2E, Additional file 1: Fig. S9A).

In summary, miR-129-5p exerts tumor-suppressive functions not only in vitro but also in vivo*.* The observed effects of miR-129-5p on cell proliferation, apoptosis, migration and EMT indicate a strong tumor-suppressive potential.

### *HDGF* is a direct target gene of miR-129-5p

To identify potential miR-129-5p target genes contributing to its tumor-suppressive effects, we transiently transfected HLE cells with miR-129-5p for 48 h and identified differentially expressed genes (p < 0.5, FC ≥ 2) by microarray analysis (Additional file 1: Fig. S4A). Downregulated genes were intersected with predicted target genes from two target gene prediction databases (TargetScan [29] and miRanda [38]) (Additional file 1: Fig. S4B). Amongst others, *hepatoma-derived growth factor* (*HDGF)* was repressed after miR-129-5p transfection (Additional file 1: Fig. S4C). We further analyzed *HDGF* since it exhibits mitogenic activity and is highly expressed in a variety of cancers [39].

**Fig. 3:**
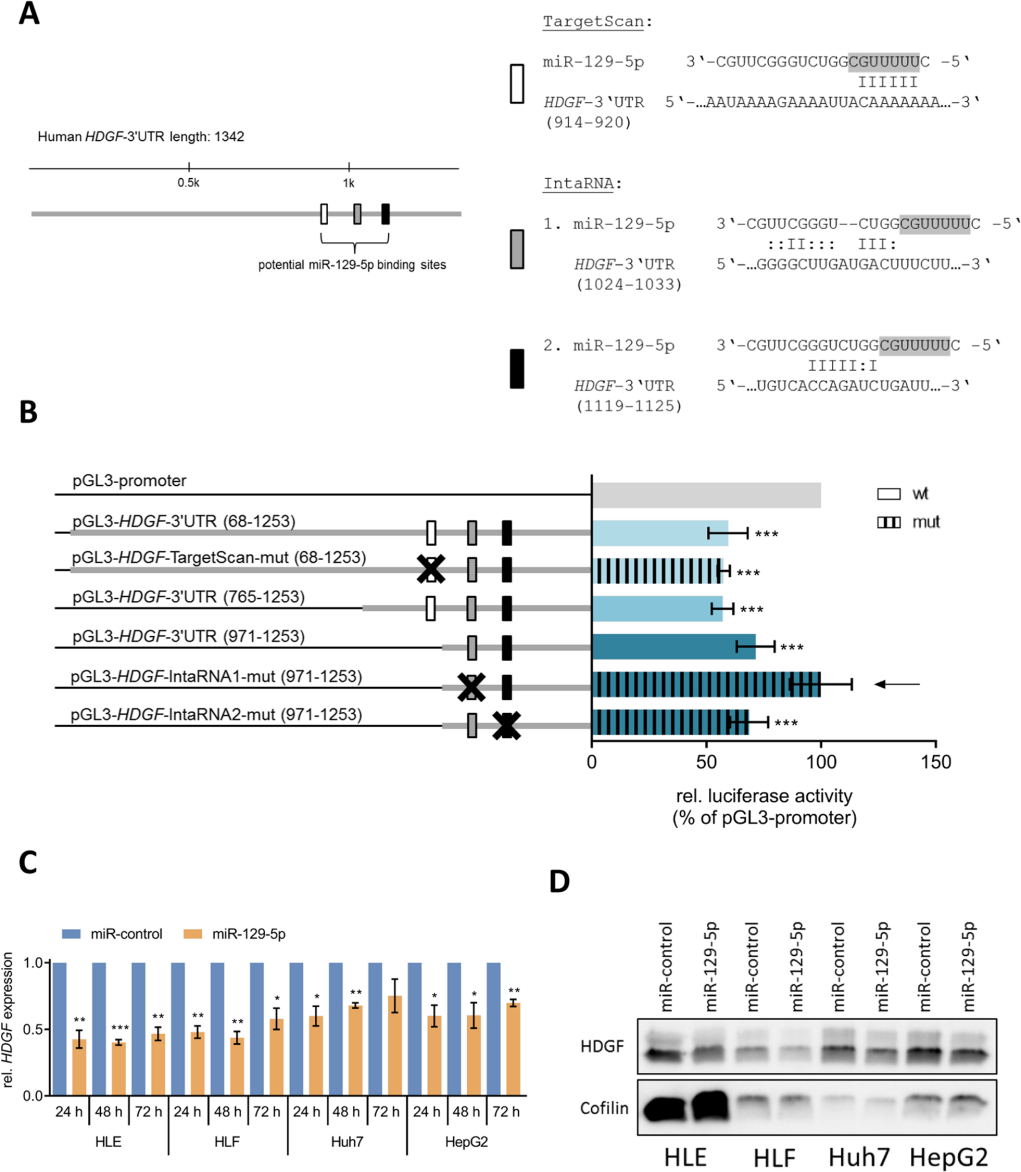
*HDGF* is a direct target gene of miR-129-5p. **A** Represented are predicted binding sites of miR-129-5p in the 3′UTR of HDGF. Binding sites were predicted by TargetScan [29] and IntaRNA [30]. The seed region of miR-129-5p is highlighted in gray. **B** Firefly luciferase activity was measured and normalized to renilla luciferase activity. ***p < 0.001; one-way ANOVA with Dunnett’s multiple comparisons test. **C** *HDGF* expression was analyzed 24 h, 48 h, and 72 h after transfection of HCC cell lines with 50 nM miR-129-5p mimics by qRT-PCR using the ΔΔCT method. *p < 0.05, **p < 0.01, ***p < 0.001; two-tailed Student’s t test. **D** HDGF protein expression was analyzed 48 h after miR-129-5p transfection by western blotting with cofilin as loading control. Densitometric analysis of western blot assays is shown in Additional file 1: Fig. S9B

Next, we performed luciferase reporter assays to validate the predicted direct regulation of *HDGF* by miR-129-5p. To achieve this, we used vectors that contain the 3′UTR of *HDGF* including either the intact or mutated binding site for miR-129-5p predicted by TargetScan (Fig. 3A). Cotransfection of miR-129-5p with these vectors, pGL3-*HDGF*-3′UTR_68–1253_ or pGL3-*HDGF*-TargetScan-mut_68–1253_, significantly reduced luciferase activity compared to the empty pGL3-promoter vector (Fig. 3B) excluding the latter as a possible binding site. To identify the so far unknown miR-129-5p binding site in the 3′UTR of *HDGF* we constructed another two vectors containing smaller fragments of the *HDGF*-3′UTR. Upon cotransfection with miR-129-5p, luciferase activity of both vectors was still significantly reduced revealing that the correct binding site was located in the smallest *HDGF*-3′UTR fragment. In this fragment (*HDGF*-3′UTR_971–1253_), two possible binding sites without involvement of the miR-129-5p seed sequence were identified by IntaRNA [30], an online tool that analyzes RNA-RNA interactions and is less restrictive regarding non-seed binding (Fig. 3A). After mutating these binding sites, cotransfection of miR-129-5p with pGL3-*HDGF*-IntaRNA1-mut_971–1253_ demonstrated no change of luciferase activity compared to the empty pGL3-promoter vector identifying IntaRNA1 as the exact binding site of miR-129-5p (Fig. 3B). This direct binding of miR-129-5p to the *HDGF* mRNA was confirmed by Ago2 immunoprecipitation (Additional file 1: Fig. S5).

In addition, we analyzed *HDGF* expression after miR-129-5p transfection or after HDACi-mediated induction of miR-129-5p. Transfection of miR-129-5p significantly reduced *HDGF* mRNA levels in all four tested HCC cell lines (Fig. 3C). This downregulation was also observed on protein level (Fig. 3D). HDAC inhibition by SAHA or FK228 significantly reduced *HDGF* mRNA levels in HCC and normal liver cell lines (Additional file 1: Fig. S6A) demonstrating the global effect of HDACi on different cell types [40]. Due to HDGF protein half-life of 120 h, the effect was less pronounced on protein level (Additional file 1: Fig. S6B, C). Together our results confirm *HDGF* as a direct target gene of miR-129-5p, which is regulated via a noncanonical binding site without seed interaction.

### Overexpression of *HDGF* correlates with a poor survival prognosis of HCC patients

**Fig. 4:**
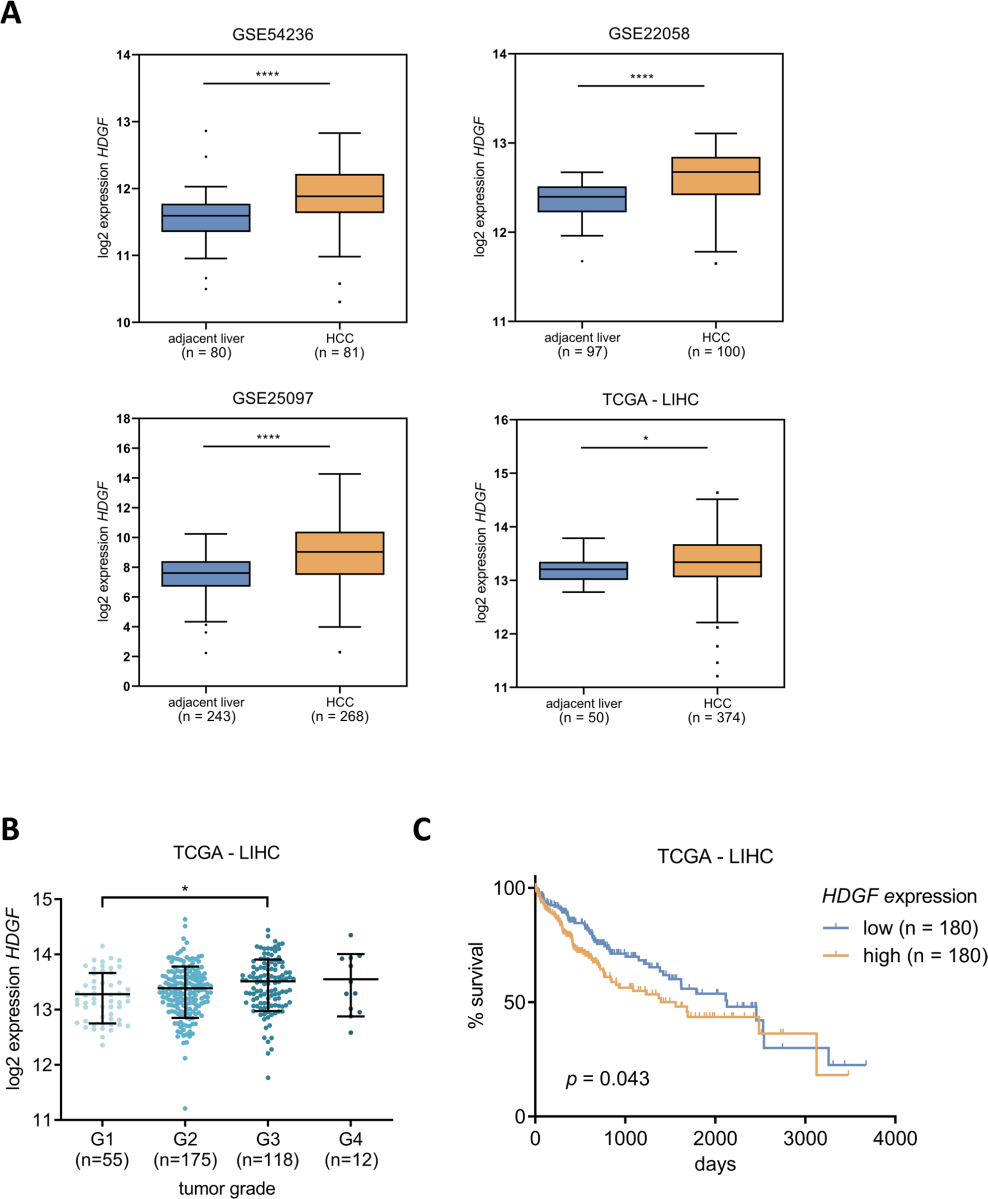
Overexpression of *HDGF* correlates with poor survival of HCC patients. **A** Expression levels of *HDGF* were analyzed using four public HCC data sets (GSE54236, GSE22058, GSE25097, TCGA-LIHC). *HDGF* expression was significantly higher in HCC tissue than in adjacent non-tumorous liver tissue. Tukey box-and-whisker plot. *p < 0.05, ****p < 0.0001; two-tailed Student’s t test. **B** *HDGF* expression levels of the TCGA-LIHC cohort were divided according to histologic tumor grade. *p < 0.05; one-way ANOVA with Tukey’s multiple comparisons test. **C** Expression values of *HDGF* and survival data of the TCGA-LIHC cohort were retrieved from OncoLnc [32]. Patients were grouped into low (lower median) or high (upper median) *HDGF* expression levels. Kaplan–Meier with log-rank test

Four publicly available HCC expression datasets were analyzed in order to determine the role of *HDGF* in hepatocellular carcinoma in vivo. A significant increase of *HDGF* expression in liver cancer tissue compared to adjacent non-tumorous liver tissue was observed in all four HCC datasets (Fig. 4A). In addition, we identified an increased *HDGF* expression in high-grade compared to low-grade HCCs (Fig. 4B). Patients with high *HDGF* expression had a worse survival prognosis compared to those with low *HDGF* expression (Fig. 4C). This indicates that *HDGF* is frequently overexpressed in HCC and that the expression of *HDGF* is particularly high in dedifferentiated HCC. Therefore, *HDGF* repression may be beneficial especially in patients with advanced HCC.

### *HDGF* knockdown exerts distinct tumor-suppressive functions in Wnt-inactive HCC cells

**Fig. 5:**
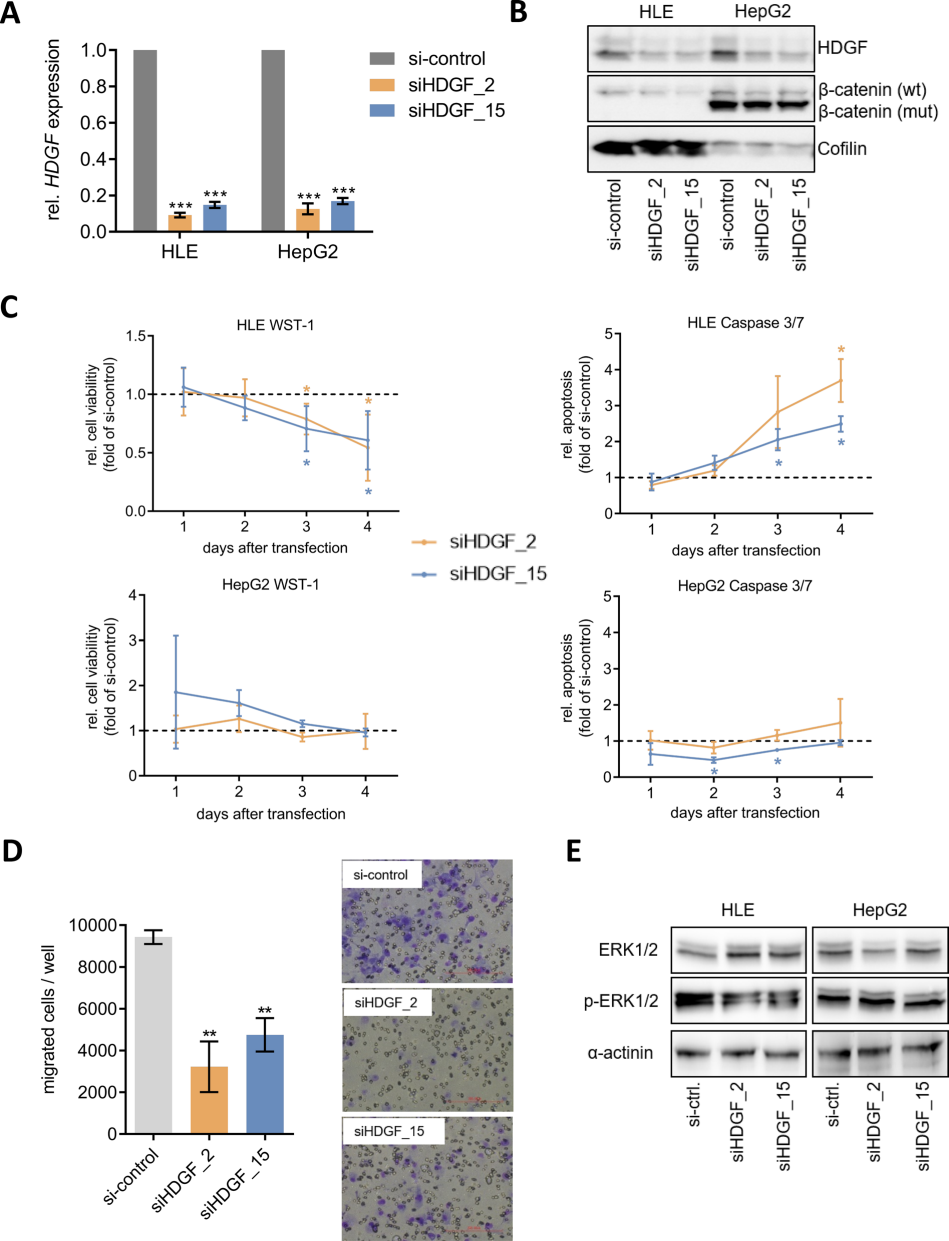
*HDGF* knockdown exerts distinct tumor-suppressive effects in Wnt-inactive HCC cells. **A** HLE and HepG2 cells were transfected with 10 nM siRNA against *HDGF. HDGF* expression was analyzed 48 h after siRNA transfection by qRT-PCR. Results were normalized to si-control. ***p < 0.001; one-way ANOVA with Dunnett’s multiple comparisons test. **B** HDGF and β-catenin protein expression was determined 48 h after siRNA transfection by western blotting with cofilin as loading control. Densitometric analysis of western blot assays is shown in Additional file 1: Fig. S9C. **C** HLE and HepG2 cells were transfected with 10 nM siRNA. Cell viability was analyzed by WST-1 assay and normalized to si-control (dotted line). Apoptosis was analyzed by Caspase 3/7 assay and normalized to cell viability and si-control (dotted line). *p < 0.05; two-way ANOVA with Dunnett’s multiple comparisons test. **D** Migration capacity of HLE cells was analyzed by transwell assay. **p < 0.01; one-way ANOVA with Dunnett’s multiple comparisons test; Scale bar = 200.00 µm. **E** HLE and HepG2 cells were transfected with 10 nM siRNA against *HDGF*. 48 h after transfection, protein expression of p-ERK1/2 and ERK1/2 was analyzed by western blotting with α-actinin as loading control. Gels were processed in parallel. Densitometric analysis of western blot assays is shown in Additional file 1: Fig. S9D

We next sought to analyze the impact of *HDGF* downregulation on tumor-relevant functions by performing cell viability and apoptosis measurements after *HDGF* knockdown. Therefore, liver cancer cell lines HLE, HLF, HepG2, and Huh6 were transiently transfected with siRNAs against *HDGF*. Successful knockdown of *HDGF* was verified by qRT-PCR (Fig. 5A, Additional file 1: Fig. S7A) and western blotting (Fig. 5B, Additional file 1: Fig. S7B). The HCC cell line HepG2 carries a deletion of 116 amino acids in exons 3–4 in *CTNNB1* (Fig. 5B) and Huh6 harbors an activating β-catenin mutation, both, resulting in constitutive activation of the Wnt/β-catenin signaling pathway [41, 42], whereas there is no detectable Wnt signaling in HLE and HLF cells [43, 44]. The cell line Hep3B harbors a mutation in *Axin1* but shows only weak Wnt signaling activity in TCF reporter assays [45], so that we did not investigate it further. Knockdown of *HDGF* decreased cell viability and significantly increased apoptosis in Wnt-inactive HLE and HLF cells (Fig. 5C, Additional file 1: Fig. S7C). In contrast, knockdown of *HDGF* did not affect cell viability and apoptosis in Wnt-active HepG2 and Huh6 cells (Fig. 5C, Additional file 1: Fig. S7C). Furthermore, *HDGF* knockdown significantly attenuated the migratory capacity of HLE and HLF cells by more than 50% compared to siRNA-control (Fig. 5D, Additional file 1: Fig. S7D). Transwell assays were not performed in HepG2 and Huh6 cells, as there is no migration detectable in these cell lines (Additional file 1: Fig. S8) [15]. To further elucidate the influence of *HDGF* knockdown on the regulation of ERK signaling, western blot analyses were performed (Fig. 5E, Additional file 1: Fig. S7E). In this study, knockdown of *HDGF* led to a reduced phosphorylation of ERK 1/2 in HLE and HLF cells, whereas protein levels of phosphorylated ERK 1/2 were not affected by *HDGF* knockdown in HepG2 and Huh6 cells (Fig. 5E, Additional file 1: Fig. S7E). This demonstrates that knockdown of *HDGF* has tumor-suppressive effects in Wnt-inactive HCCs. The Wnt status-dependent, tumor-suppressive role of *HDGF* knockdown may lead to the urgently needed targeted and personalized HCC therapy.

### High levels of *HDGF* correlate with poor survival in HCCs with inactive Wnt signaling

**Fig. 6:**
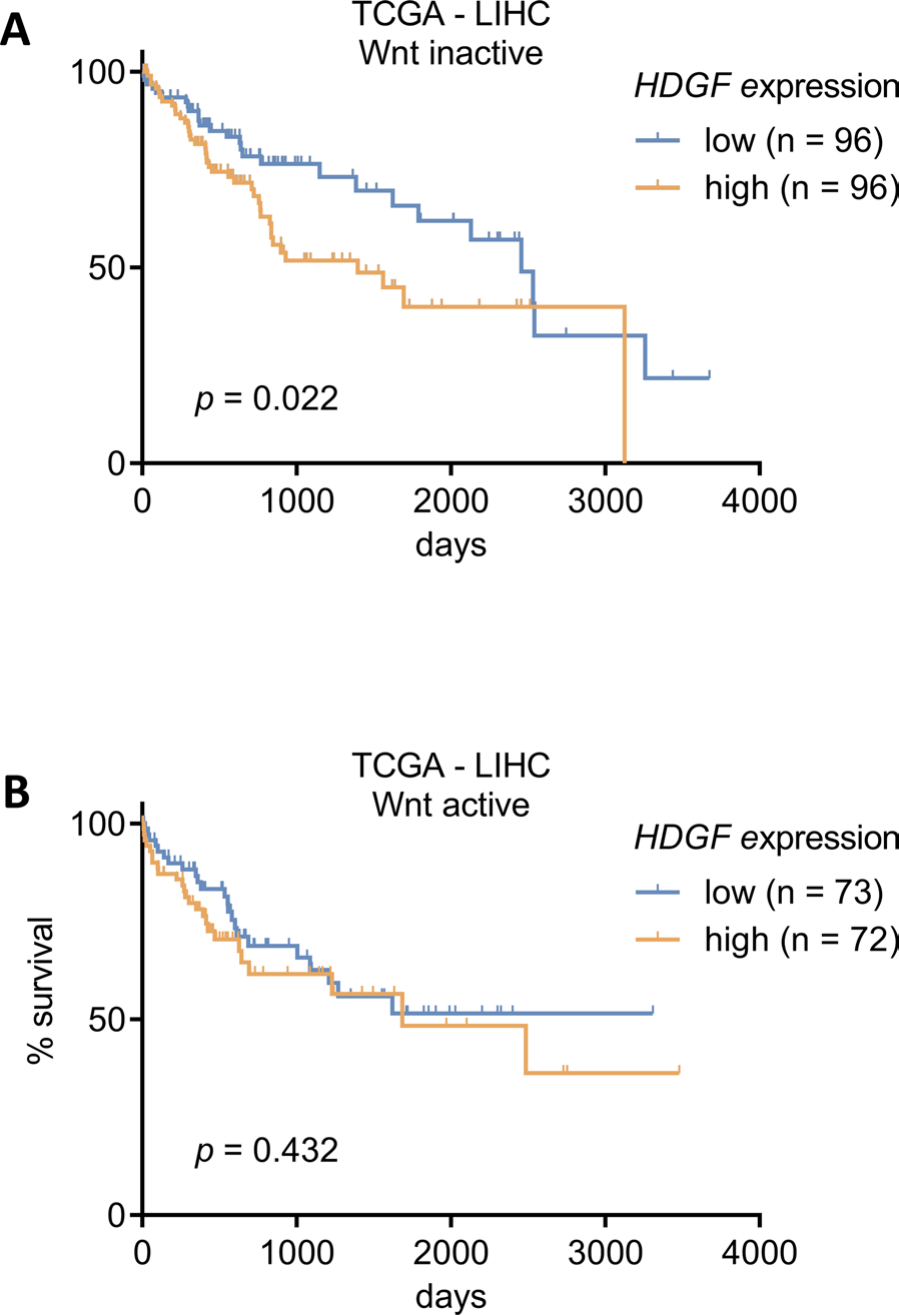
High levels of *HDGF* correlate with poor survival of patients in HCCs with inactive Wnt signaling. **A**, **B** *HDGF* expression values and survival data of the TCGA-LIHC cohort were retrieved from OncoLnc [32] and stratified into Wnt-inactive (**A**) and Wnt-active (**B**) HCCs according to Sanchez-Vega et al*.* [33]. Patients were grouped into low (lower median) or high (upper median) expressions of *HDGF*. Kaplan–Meier with log-rank test

Our in vitro results suggested a Wnt signaling status-dependent transmission of *HDGF’s* tumor-suppressive effects in HCC cell lines. This led us to reanalyze the survival data of the TCGA-LIHC cohort taking the Wnt signaling status of HCCs into account. Therefore, we stratified the TCGA-LIHC cohort into Wnt-active and Wnt-inactive HCCs according to Sanchez-Vega et al. [33]. The analyses showed a correlation of high *HDGF* expression with poor overall survival especially in Wnt-inactive HCCs (Fig. 6). Together, our results confirm the significant, oncogenic role of *HDGF* in Wnt-inactive HCCs.

## Discussion

In this study, we comprehensively characterized miR-129-5p and its direct target gene *HDGF* in the context of hepatocellular carcinoma. Specifically, we investigated whether miR-129-5p is a suitable candidate for a miRNA replacement therapy in HCC since modulating miRNA expression is a promising approach for personalized medicine against cancers. Our study provides a link between the tumor-suppressive miR-129-5p and the mitogenic growth factor *HDGF* that is frequently overexpressed in HCC. We suggest that HCC patients of the Wnt-inactive subgroup may benefit from targeting *HDGF*, for example by the therapeutic use of miR-129-5p.

The role of microRNAs in tumorigenesis has come into focus by our previous studies showing the epigenetic deregulation in HCC of miR-129-5p and several other microRNAs [8]. Disruption of the epigenetic machinery, for example by overexpression of HDACs, as has been observed in HCC [7–9], contributes to tumor development and its progression through silencing of tumor-suppressive miRNAs. Therefore, modulating miRNA expression by HDACi targeting these epigenetic modifiers is a promising approach for cancer therapy. In this study, we demonstrate that the treatment of HCC cell lines with HDAC inhibitors induces the expression of several miRNAs, among them miR-129-5p. In addition, we show that miR-129-5p inhibits proliferation, attenuates migration and induces apoptosis in vitro and suppresses tumor growth in vivo in hepatocellular carcinoma. Similar tumor-suppressive effects of miR-129-5p have also been observed in osteosarcoma [46], prostate [47] and breast cancer [48].

The downregulation of miR-129-5p in HCC and other tumor entities is usually attributable to a miR-129-2 promoter hypermethylation [18–22]. Here, FDA-approved HDACi SAHA and FK228 led to the re-expression of miR-129-5p due to the induction of *pri-miR-129-2* expression in HLE cells*.* Thus, miR-129-5p may not only be epigenetically downregulated by miR-129-2 promoter hypermethylation but also by increased histone deacetylation of the miR-129-2 promoter. Since HDACi have a range of undesirable side effects and act relatively unspecific, the targeted application of tumor-suppressive miRNAs is a favorable therapeutic approach [49]. Notably, several potential miRNA therapies have reached phase I and phase II clinical trials [50, 51]. The FDA approval of patisiran [52], an siRNA for the treatment of patients with hereditary transthyretin-mediated amyloidosis, points towards the great potential of RNA interference therapeutics. However, the development of miRNA replacement therapies requires thorough characterization of candidate miRNAs, their targetomes and involved regulatory pathways.

The comprehensive characterization of microRNA target genes provides the basis for the direct therapeutic application of miRNAs. To contribute to the comprehensive characterization of miR-129-5p, we aimed to elucidate its role in migration and EMT by targeting *SOX4*. The transcription factor *SOX4* is a well-established target gene of miR-129-5p [18, 53] which has been identified as a key factor of TGF-β-mediated induction of EMT facilitating migration and metastasis [23, 24]. We have previously demonstrated that activation of the TGF-β pathway in HLE cells induces *SOX4* expression and increases their migratory capacity [15]. Here, miR-129-5p not only abrogated the TGF-β-mediated *SOX4* induction but more importantly, inhibited TGF-β-mediated cell migration of HLE and HLF cells. Transfection of HLE cells with miR-129-5p also resulted in induced expression of epithelial marker *CDH1* and decreased expression of mesenchymal marker *VIM* suggesting that miR-129-5p inhibits EMT. These results are in line with Xiao et al*.* showing that miR-129-5p suppresses TGF-β-mediated migration of human peritoneal mesothelial cells [54] and also with results from Luan et al*.* who have demonstrated that miR-129-5p regulates EMT in breast cancer cells [55]. In summary, miR-129-5p inhibits cell migration and EMT by preventing TGF-β-mediated *SOX4* overexpression.

To provide more detailed knowledge of miR-129-5p in hepatocarcinogenesis, we aimed to identify direct targets of miR-129-5p. Our study establishes a link between hepatoma-derived growth factor *HDGF* and miR-129-5p. We validated *HDGF* as a direct target gene of miR-129-5p that is downregulated via a noncanonical binding site in the *HDGF-*3′UTR. Interestingly, transcriptome-wide analyses of miRNA-mRNA interactions have demonstrated that non-seed binding sites are common and functional [56]. *HDGF* is highly expressed in a variety of cancers, which is—regardless of the tumor type—linked to a negative outcome for patients [39]. Confirming these findings, four different HCC gene expression datasets revealed *HDGF* overexpression in HCC, providing evidence for an oncogenic role of *HDGF* in HCC. Furthermore, we showed a significant correlation of high *HDGF* expression levels with a shorter overall survival of HCC patients offering potential prognostic value for *HDGF* in liver cancer. Taken together, *HDGF* appears to act as an oncogene and, therefore, its inhibition or downregulation via miR-129-5p may result in an improved HCC therapy.

Possible roles for *HDGF* in carcinogenesis are already known. *HDGF* encodes a protein member of the hepatoma-derived growth factor family that has mitogenic and DNA-binding activity and has been described to be involved in cell proliferation [57], apoptosis [58], migration [59] and upregulating the ERK/MAPK signaling pathway [60]. Consistent with these observations, we demonstrated increased apoptosis and reduced cell viability as well as mitigated migration after *HDGF* knockdown in the HCC cell lines HLE and HLF. These effects may be mediated by an inhibition of ERK1/2 signaling, since both downregulation of *HDGF* and miR-129-5p transfection led to reduced levels of phosphorylated ERK1/2 in HLE and HLF cells. These results are in line with Zeng et al*.* who have previously observed decreased levels of p-ERK1/2 upon miR-129-5p overexpression in human glioblastoma cells [36]. Thus, our results suggest that downregulation of *HDGF* contributes to the tumor-suppressive effects of miR-129-5p by inhibiting ERK1/2 signaling.

Interestingly, we observed that knockdown of *HDGF* or miR-129-5p transfection did not influence cell viability or apoptosis of Wnt-active HepG2 and Huh6 cells. In Wnt-inactive HLE and HLF cells, however, miR-129-5p and *HDGF* knockdown resulted in increased apoptosis and reduced cell viability, hinting towards Wnt status-dependent functions of miR-129-5p and its target gene *HDGF*. These in vitro results fully concur with our survival analysis of the TCGA liver cancer data set, demonstrating that high *HDGF* expression correlates with poor survival only in Wnt-inactive human HCCs but not in Wnt-active HCCs. Wnt signaling activity plays an important role in characterizing HCCs. Pinyol and Llovet et al*.* have performed molecular classification of HCC based upon immune status, resulting in two major groups [6, 61]. Around 30% of HCCs belong to the ‘Immune class’ with high levels of immune cell infiltration whereas 30% account for the ‘Immune Exclusion class’, which is characterized by *CTNNB1* mutations leading to constitutive active Wnt signaling [61]. Both HCC classes respond differentially to current therapy options [6]. However, the molecular classification of HCC needs to be further characterized to be able to treat these patients in a targeted manner. This is the first study considering the Wnt status-dependent tumor-suppressive effects of miR-129-5p and *HDGF* knockdown in HCC. Our findings demonstrate the need for the development of new personalized HCC therapies.

## Conclusions

In conclusion, we here present the first evidence that miR-129-5p and downregulation of its direct target gene *HDGF* may have significant implications for the treatment of patients with Wnt-inactive, advanced HCC. We demonstrated that mir-129-5p is inducible by epigenetic drugs (i.e. HDACi) and exhibits distinct tumor-suppressive functions by downregulating *HDGF.* Therefore, our data suggest that miR-129-5p may be suitable for therapeutic application and is a promising candidate for a miRNA replacement therapy in advanced HCC. Our results contribute to the development of targeted HCC treatment strategies and emphasize the importance of an extensive characterization and detailed knowledge of regulated pathways for the development of new personalized HCC therapies.

## Supplementary Information

**Additional file 1: Figure S1.** SAHA and FK228 induce the expression of *pri-miR-129-2*. **Figure S2.** HDAC inhibition increases acetylation and apoptosis, while decreasing cell viability of HCC cell lines and normal liver cell lines. **Figure S3.** miR-129-5p prohibits TGF-β-mediated *SOX4* overexpression and cell migration. **Figure S4.** *HDGF* is a target of miR-129-5p. **Figure S5.** *HDGF* is enriched in RISCs after miR-129-5p transfection. **Figure S6.** HDAC inhibition reduces *HDGF* expression. **Figure S7.** *HDGF* knockdown exerts distinct tumor-suppressive effects in Wnt-inactive HCC cells. **Figure S8.** Migration capacity of HepG2, Huh6, and Huh7 cells. **Figure S9.** Densitometric analysis of western blot assays. **Table S1.** Primer for cloning of luciferase reporter vectors. **Table S2.** siRNAs and miRNA mimic for transfection. **Table S3.** gBlocks gene fragments (IDT). **Table S4.** TaqMan Assays for quantitative real-time PCR. **Table S5.** Antibodies for AGO2-IP.

## Data Availability

The public datasets analyzed during the current study are available in the repositories listed below: *Gene Expression Omnibus*—GSE22058: https://www.ncbi.nlm.nih.gov/geo/query/acc.cgi?acc=GSE22058. GSE25097: https://www.ncbi.nlm.nih.gov/geo/query/acc.cgi?acc=GSE25097. GSE54236: https://www.ncbi.nlm.nih.gov/geo/query/acc.cgi?acc=GSE54236. *The Cancer Genome Atlas*—TCGA-LIHC: https://portal.gdc.cancer.gov/projects/TCGA-LIHC. *OncoLnc*—OncoLnc-LIHC: http://www.oncolnc.org/. MicroRNA sequencing data generated during this study are available in the NCBI GEO database under the GEO accession GSE182460 (https://www.ncbi.nlm.nih.gov/geo/). Microarray data are available from the corresponding author upon reasonable request.

## Abbreviations

AGO2-IP: Argonaute-2 immunoprecipitation
EMT: Epithelial to mesenchymal transition
FCS: Fetal calf serum
HCC: Hepatocellular carcinoma
HDAC: Histone deacetylase
HDACi: Histone deacetylase inhibitor
HDGF: Hepatoma-derived growth factor
miRNA: MicroRNA
p-ERK: Phosphorylated ERK
RISC: RNA-induced silencing complex
SAHA: Suberoyl anilide hydroxamic acid
siRNA: Small interfering RNA
SOX4: SRY (sex determining region Y)-box 4
TCGA-LIHC: The Cancer Genome Atlas-Liver Hepatocellular Carcinoma
TGF-β: Transforming growth factor-β
TSA: Trichostatin A
3′UTR: 3′ Untranslated region
