# Supplementary material for: MiR-129-5p exerts Wnt signaling-dependent tumor-suppressive functions in hepatocellular carcinoma by directly targeting hepatoma-derived growth factor HDGF
Source: Cancer Cell Int. 2022 May 16;22:192. doi: 10.1186/s12935-022-02582-2 (PMC9109340; doi:10.1186/s12935-022-02582-2)
Supplement: Supplementary file 1 — Additional file 1: Figure S1. SAHA and FK228 induce the expression of pri-miR-129-2. Figure S2. HDAC inhibition increases acetylation and apoptosis, while decreasing cell viability of HCC cell lines and normal liver cell lines. Figure S3. miR-129-5p prohibits TGF-β-mediated SOX4 overexpression and cell migration. Figure S4. HDGF is a target of miR-129-5p. Figure S5. HDGF is enriched in RISCs after miR-129-5p transfection. Figure S6. HDAC inhibition reduces HDGF expression. Figure S7. HDGF knockdown exerts distinct tumor-suppressive effects in Wnt-inactive HCC cells. Figure S8. Migration capacity of HepG2, Huh6, and Huh7 cells. Figure S9. Densitometric analysis of western blot assays. Table S1. Primer for cloning of luciferase reporter vectors. Table S2. siRNAs and miRNA mimic for transfection. Table S3. gBlocks gene fragments (IDT). Table S4. TaqMan Assays for quantitative real-time PCR. Table S5. Antibodies for AGO2-IP. [file 12935_2022_2582_MOESM1_ESM.docx]

**Additional file 1**

**MiR-129-5p exerts Wnt signaling-dependent tumor-suppressive functions in hepatocellular carcinoma by directly targeting hepatoma-derived growth factor *HDGF***

Nicole Huge^CO^, Thea Reinkens^CO^, Reena Buurman, Maria Sandbothe, Anke Bergmann, Hannah Wallaschek, Beate Vajen, Amelie Stalke, Melanie Decker, Marlies Eilers, Vera Schäffer, Oliver Dittrich-Breiholz, Engin Gürlevik, Florian Kühnel, Brigitte Schlegelberger, Thomas Illig, Britta Skawran

[Additional figures 2](#_Toc96594859)

[Figure S1 SAHA and FK228 induce the expression of *pri-miR-129-2* 2](#_Toc96594860)

[Figure S2 HDAC inhibition increases acetylation and apoptosis, while decreasing cell viability of HCC cell lines and normal liver cell lines. 3](#_Toc96594861)

[Figure S3 miR-129-5p prohibits TGF-β-mediated *SOX4* overexpression and cell migration 4](#_Toc96594862)

[Figure S4 *HDGF* is a target of miR-129-5p 5](#_Toc96594863)

[Figure S5 *HDGF* is enriched in RISCs after miR-129-5p transfection 6](#_Toc96594864)

[Figure S6 HDAC inhibition reduces *HDGF* expression 7](#_Toc96594865)

[Figure S7 *HDGF* knockdown exerts distinct tumor-suppressive effects in Wnt-inactive HCC cells 8](#_Toc96594866)

[Figure S8 Migration capacity of HepG2, Huh7 and, Huh6 cells 10](#_Toc96594868)

[Figure S9 Densitometric analysis of western blot assays 12](#_Toc96594873)

[Tables 13](#_Toc96594874)

[Table S1 Primer for cloning of luciferase reporter vectors 13](#_Toc96594875)

[Table S2 siRNAs and miRNA mimic for transfection 13](#_Toc96594876)

[Table S3 gBlocks gene fragments (IDT) 13](#_Toc96594877)

[Table S4 TaqMan Assays for quantitative real-time PCR 14](#_Toc96594878)

[Table S5 Antibodies for AGO2-IP 14](#_Toc96594879)

Additional figures


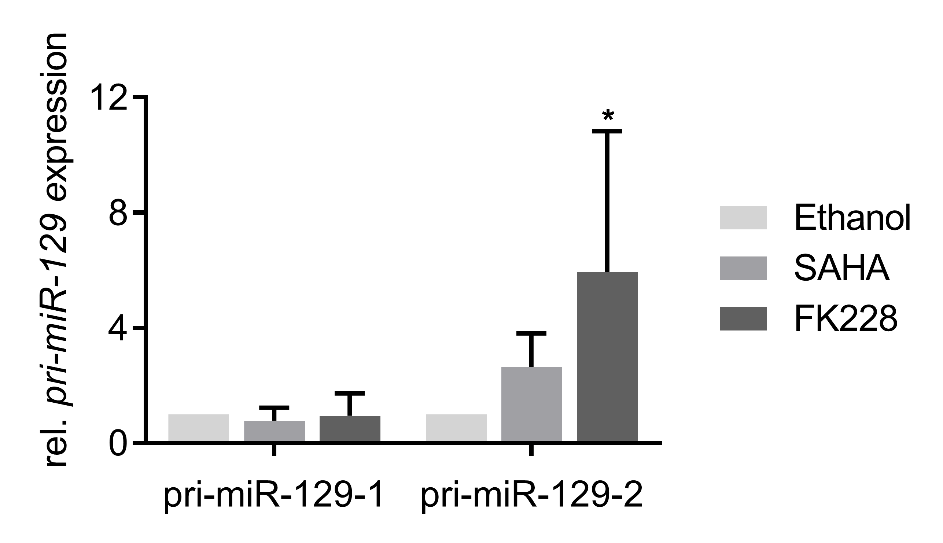


Figure S1 SAHA and FK228 induce the expression of *pri-miR-129-2*

HLE cells were treated with 2 µM SAHA or 35 nM FK228 or ethanol vehicle control for 24 h. Expression of *pri-miR-129-1* and *pri-miR-129-2* were determined by qRT-PCR using the ΔΔCT method. Data are represented as mean ± SD of three independent experiments. *p < 0.05; two-tailed Student’s *t* test.

**A**

**B**

**C**


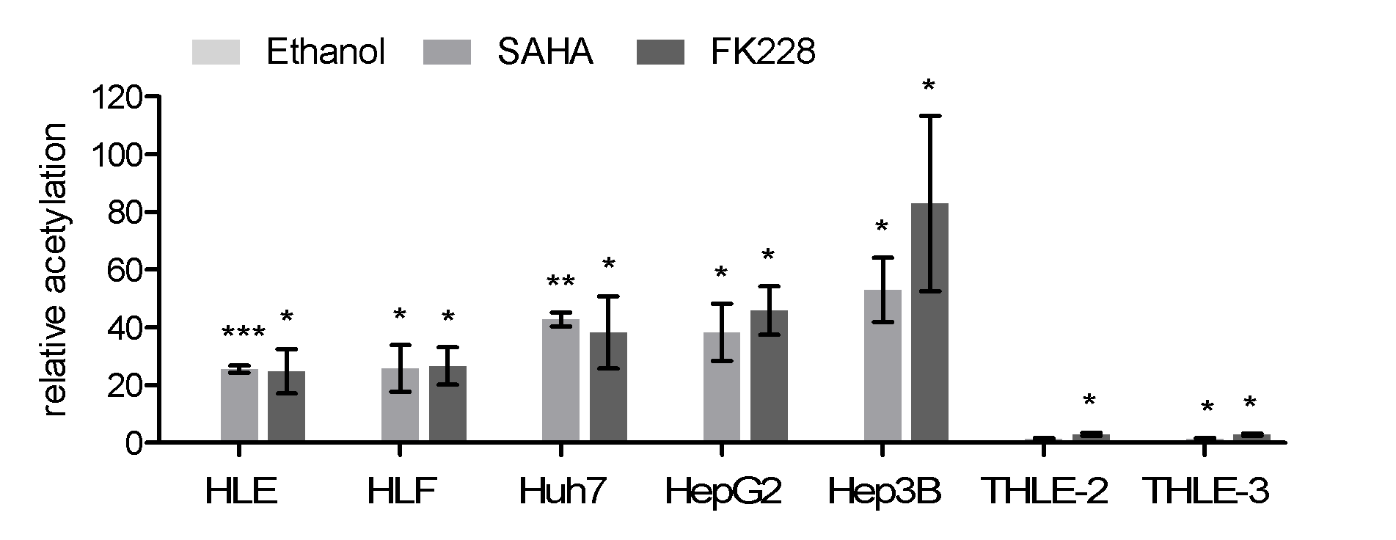

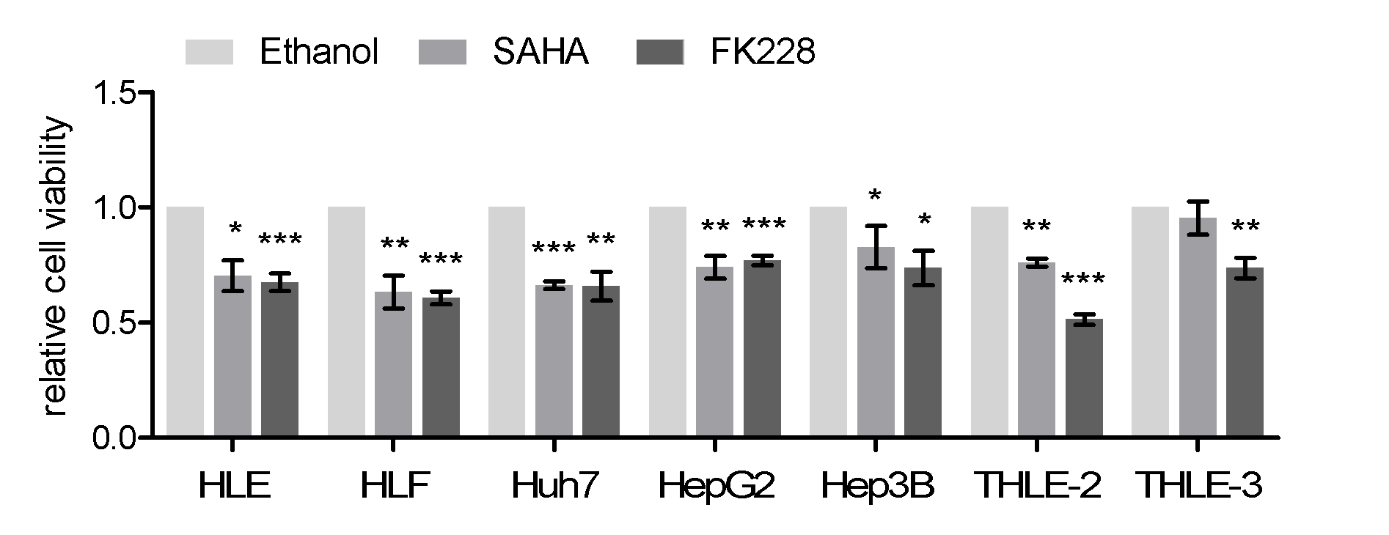

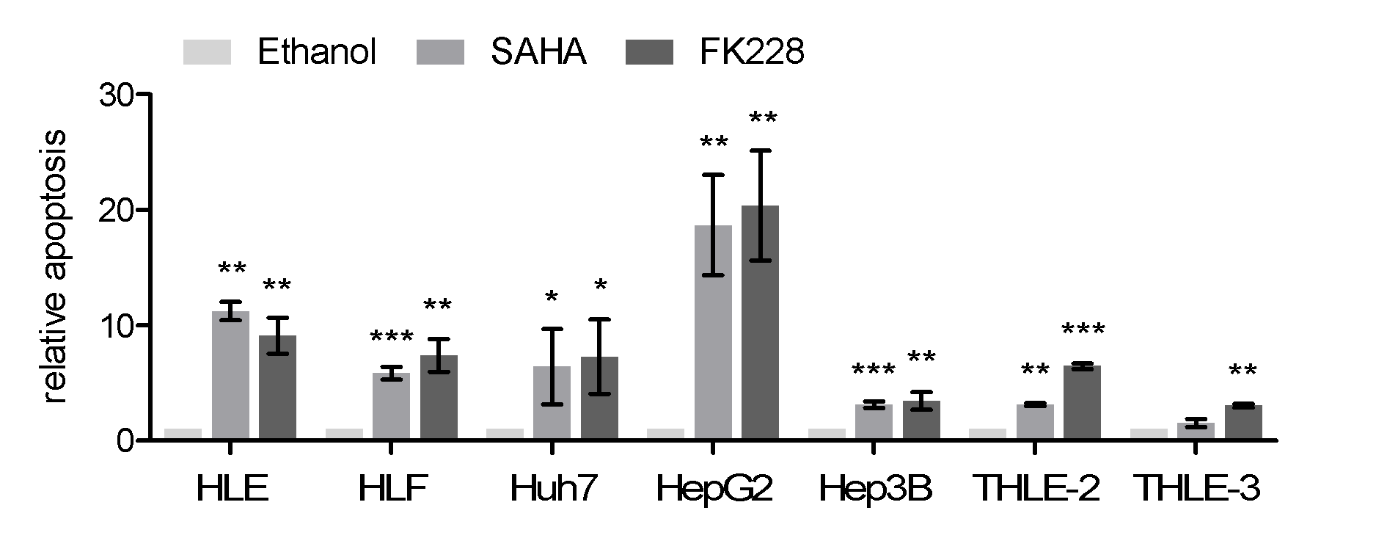


Figure S2 HDAC inhibition increases acetylation and apoptosis, while decreasing cell viability of HCC cell lines and normal liver cell lines.

Global histone acetylation (**A**), cell viability (**B**), and apoptosis (**C**) of HCC and normal liver cell lines were analyzed after 24 h treatment with 2 µM SAHA or 35 nM FK228 or ethanol vehicle control. Cell viability was normalized to ethanol treated cells. Acetylation and apoptosis were normalized to cell viability and ethanol treated cells. Values of ethanol treated control cells were set to 1 to enable a better comparability of the data. Data are represented as mean ± SD of three independent experiments. *p < 0.05, **p < 0.01, ***p < 0.001; two-tailed Student’s *t* test.


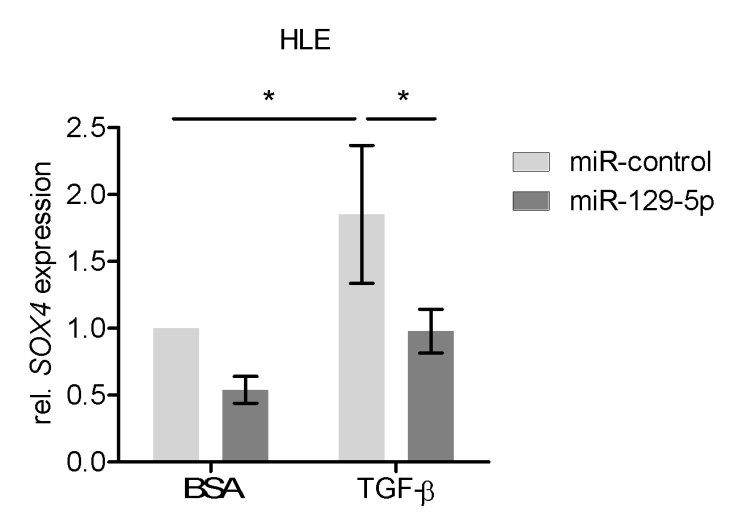

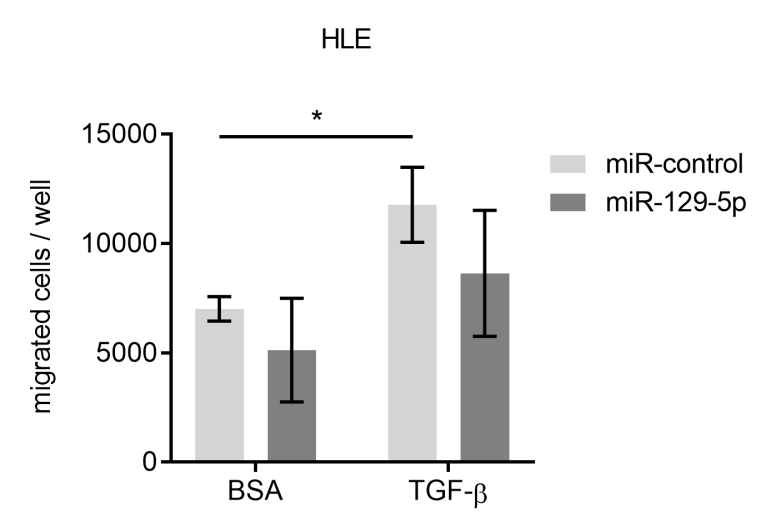

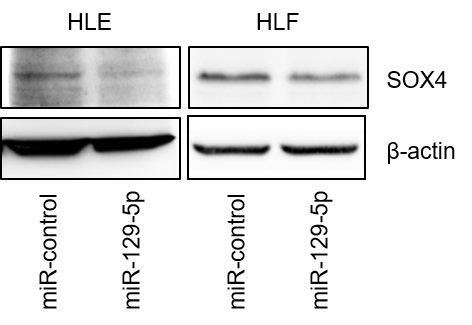

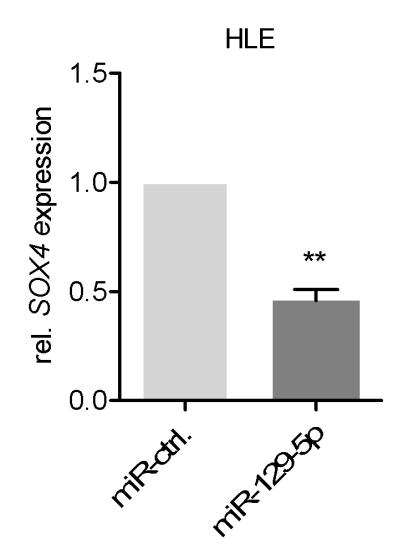

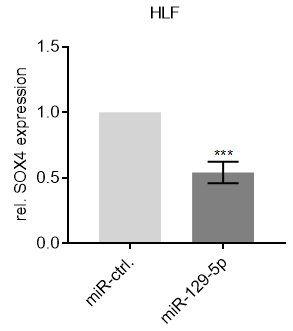

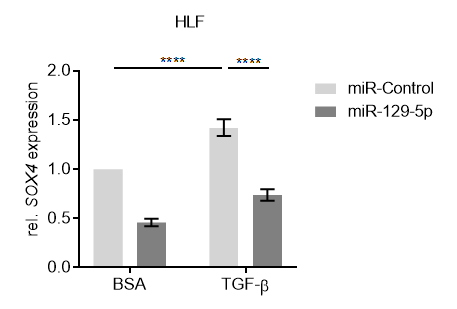


**A**

**B**

**C**

**D**


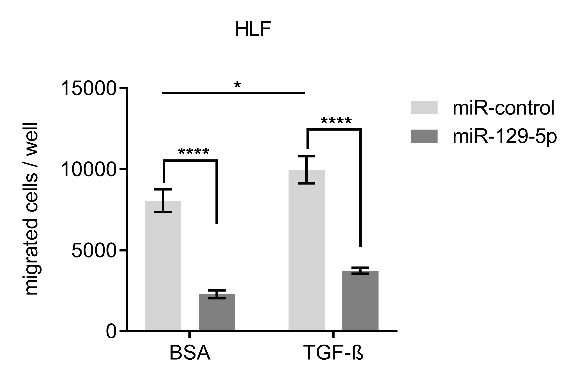


Figure S3 miR-129-5p prohibits TGF-β-mediated *SOX4* overexpression and cell migration

(**A**) *SOX4* expression was analyzed 48 h after transfection of HLE and HLF cells with 50 nM miR-129-5p mimics by qRT-PCR using the ΔΔCT method. Data are represented as mean ± SD of three independent experiments. **p < 0.01, ***p < 0.001; two-tailed Student’s *t* test. (**B**) SOX4 protein expression was determined by western blot with β-actin as loading control. Densitometric analysis of western blot assays is shown in Fig. S9E.

(**C+D**) HLE and HLF cells were transfected with 50 nM miRNA mimics and after 24 h cells were treated with 5 ng/mL TGF-β or bovine serum albumin (BSA) vehicle control. (**C**) 72 h after TGF-β treatment, *SOX4* expression was analyzed by qRT-PCR and normalized to miR-Control/BSA-treated cells. (**D**) 24 h after TGF-β treatment, cell migration was analyzed by transwell assays. Data are represented as mean ± SD of three independent experiments. *p < 0.05, ****p < 0.0001; one-way ANOVA with Tukey’s multiple comparison test.

**A**

**B**

**C**

miR-control

miR-129-5p


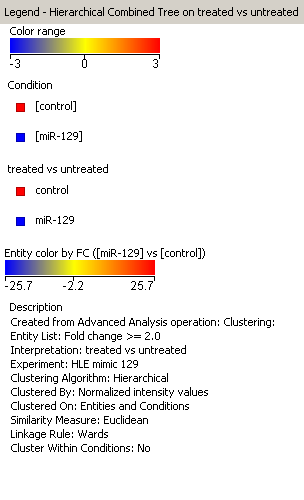

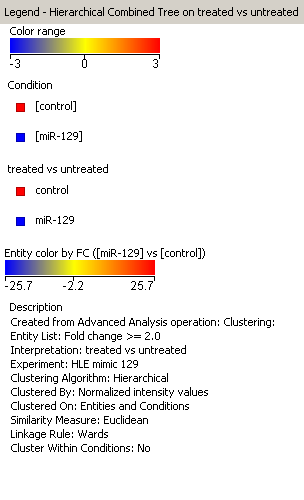

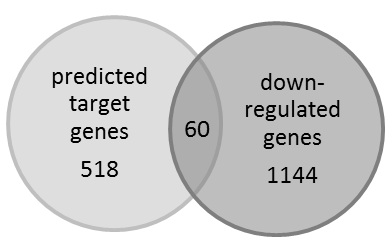

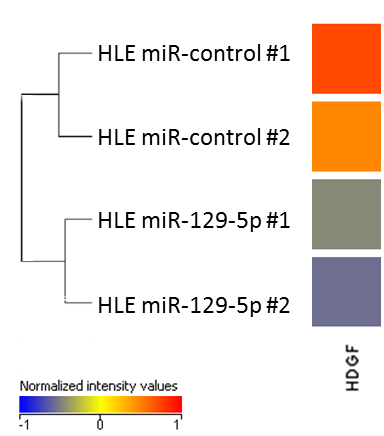

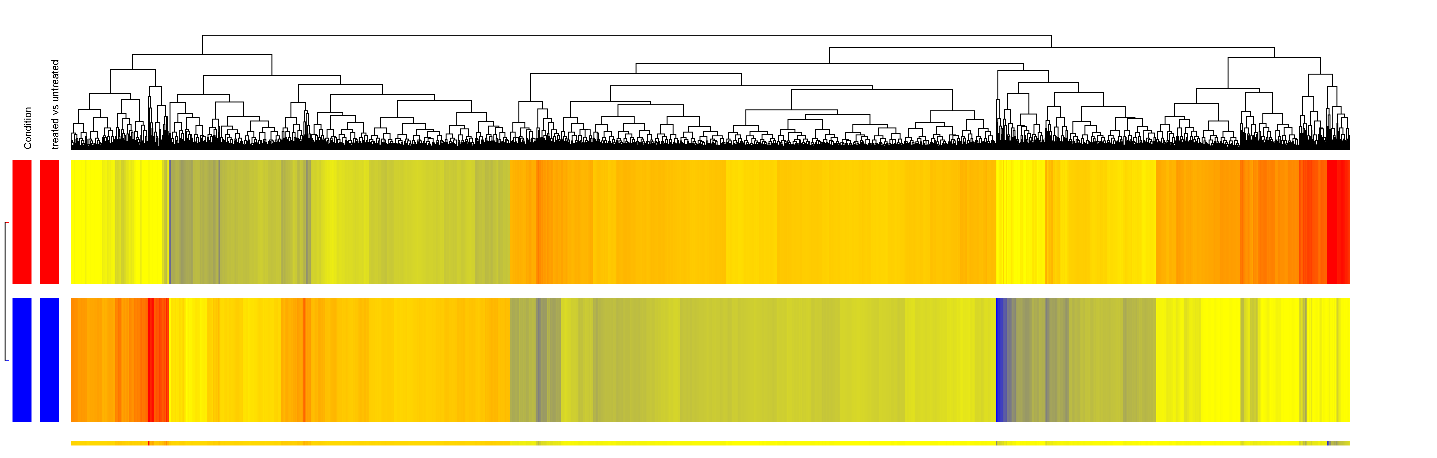


Figure S4 *HDGF* is a target of miR-129-5p

(**A**) Differentially expressed genes (p < 0.5, two-tailed Student’s *t* test, FC ≥ 2) were determined by global mRNA expression profiling after transfection of HLE cells with 50 nM miR-129-5p mimics for 48 h.

(**B**) Downregulated genes (p < 0.5, two-tailed Student’s *t* test, FC ≥ 2) determined by mRNA microarray after miR-129-5p transfection were intersected with predicted miR-129-5p target genes from two target gene prediction databases (TargetScan (29) and miRanda (38)). Thereby, among others, *HDGF* was identified as a potential miR-129-5p target gene.

(**C**) Expression of *HDGF* after miR-129-5p transfection in comparison to miR-control treated cells as determined by mRNA microarrays.

**A**

**B**

**C**


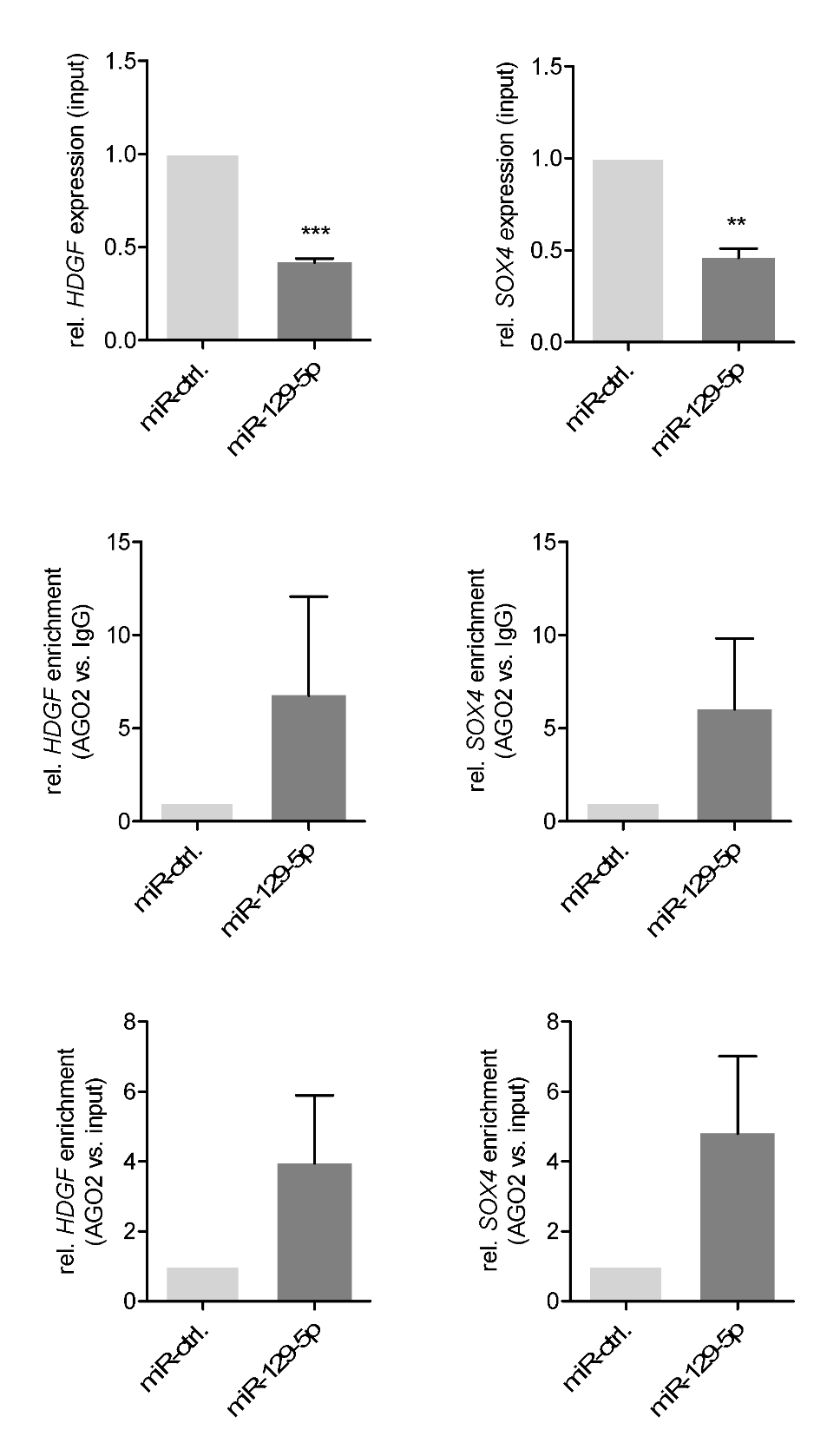


Figure S5 *HDGF* is enriched in RISCs after miR-129-5p transfection

HLE cells were transiently transfected with 50 nM miR-129-5p mimics. After 48 h cells were lysed and used as input for the AGO2-IP. (**A**) In the input, expression of *HDGF* and the positive control *SOX4* was measured by qRT-PCR using the ΔΔCT method. (**B+C**) The RNA that was isolated by AGO2-IP was used to determine the relative enrichment of *HDGF* and *SOX4* in the RISCs of miR-129-5p treated cells in comparison to miR-control treated cells. Expression was measured by qRT-PCR using the ΔΔCT method and normalized to (**B**) the IgG controls or (**C**) to the input. Data are represented as mean ± SD of three independent experiments. **p < 0.01, ***p < 0.001; two-tailed Student’s *t* test.

**A**

**B**


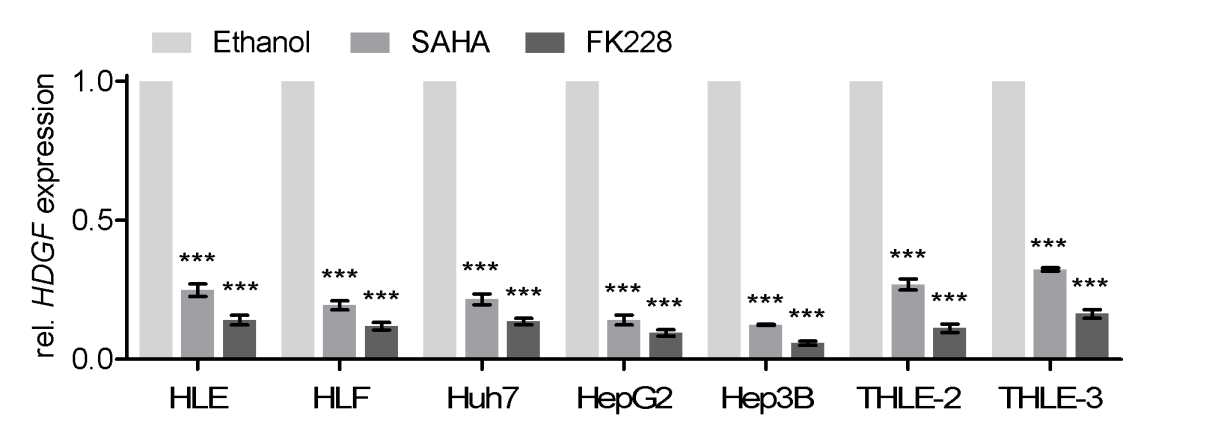

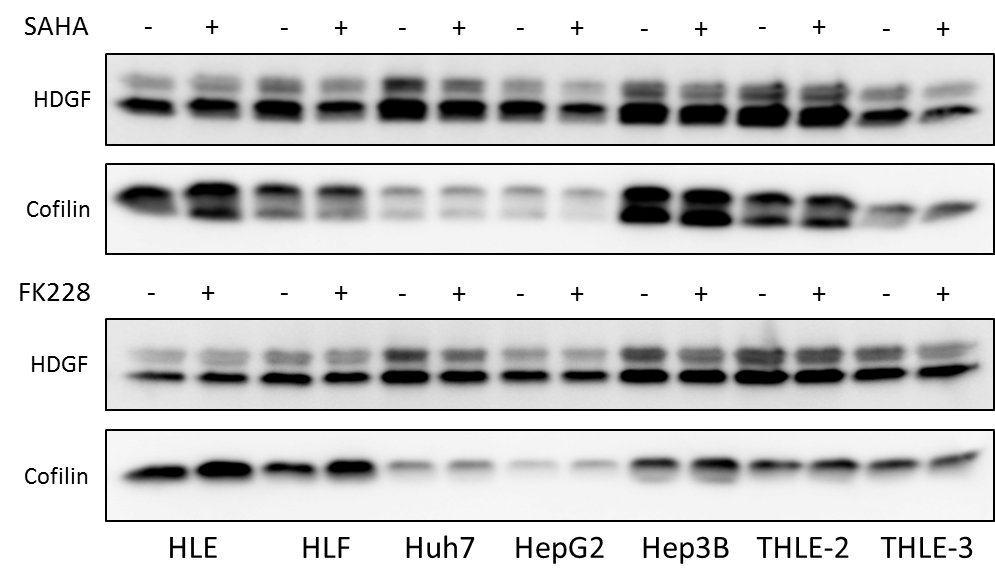

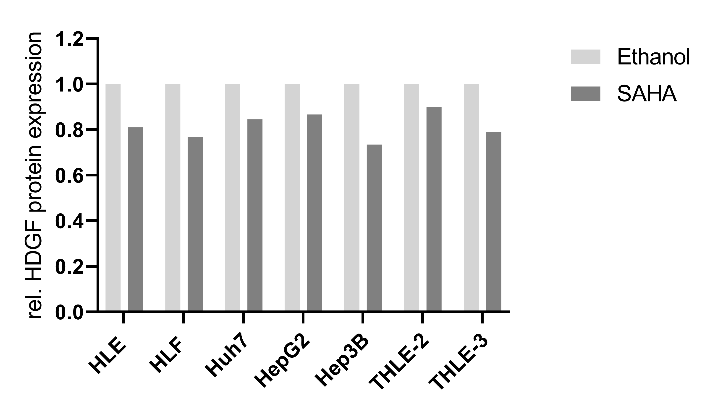

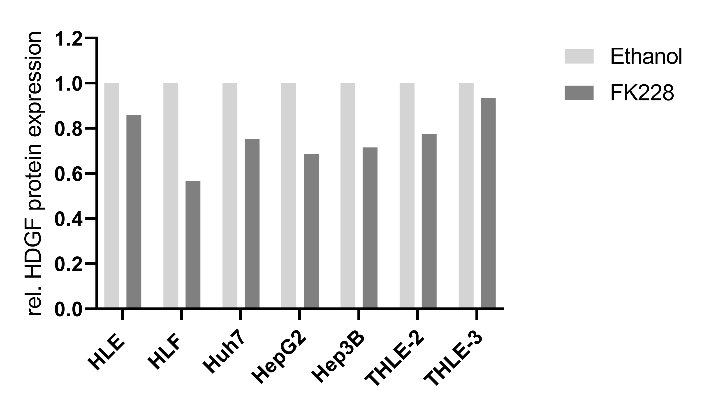


**C**

Figure S6 HDAC inhibition reduces *HDGF* expression

HCC and normal liver cell lines were treated with 2 µM SAHA or 35 nM FK228 or ethanol vehicle control for 24 h.

(**A**) *HDGF* expression was analyzed by qRT-PCR using the ΔΔCT method and normalized to ethanol treated cells. Data are represented as mean ± SD of three independent experiments. ***p < 0.001; two-tailed Student’s *t* test.

(**B**) HDGF protein expression was determined by western blot with cofilin as loading control.

(**C**) Densitometric analysis of western blot assays. HDGF protein expression was normalized to loading control.


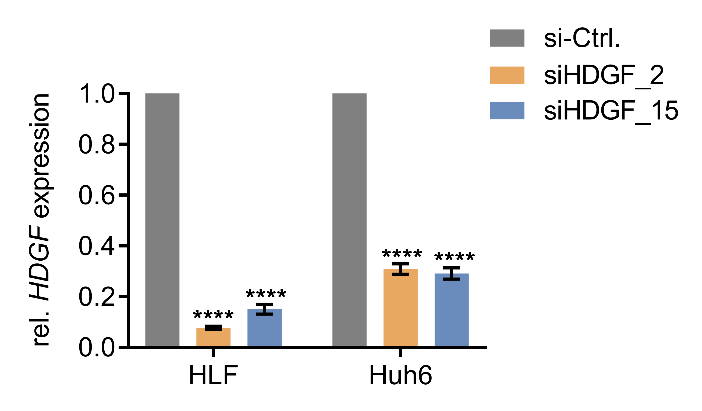

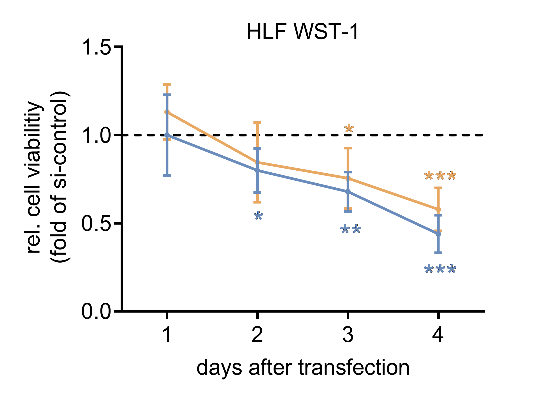

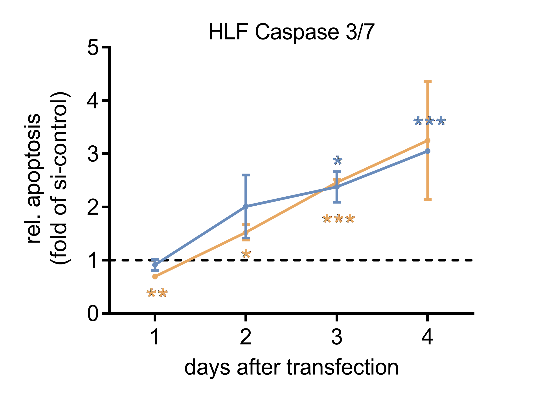

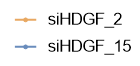

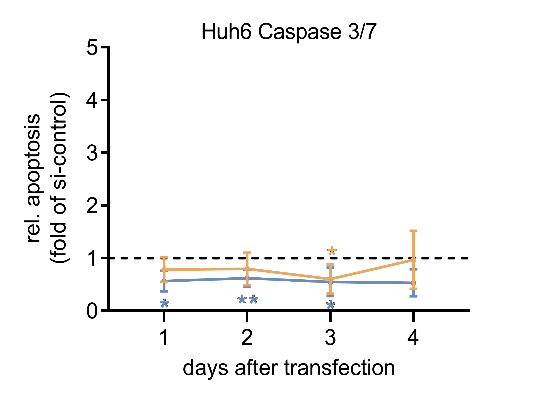

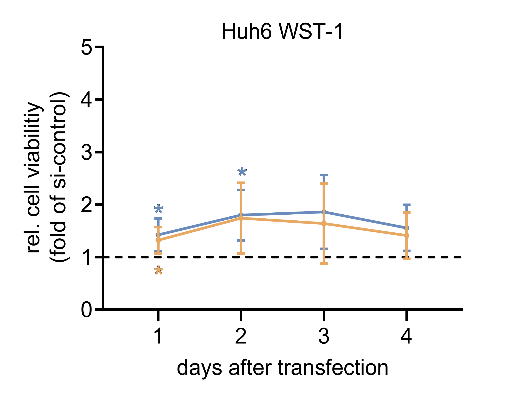

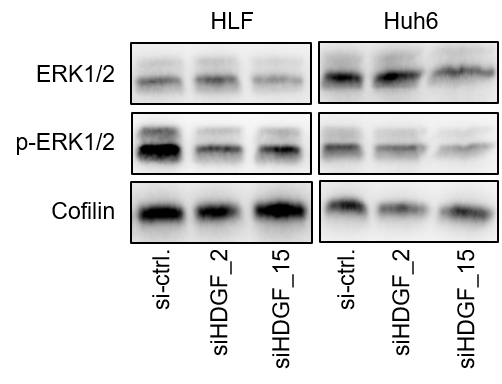


**A**

**C**

**D**

**E**


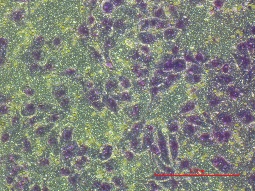

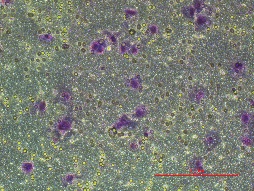

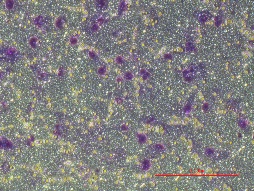


si-control

siHDGF_2

siHDGF_15


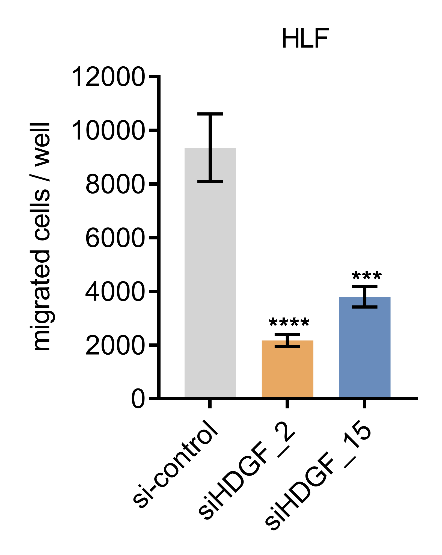


**B**


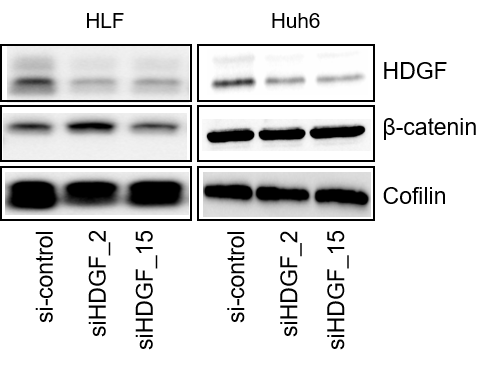


Figure S7 *HDGF* knockdown exerts distinct tumor-suppressive effects in Wnt-inactive HCC cells

**(A)** HLF and Huh6 cells were transfected with 10 nM siRNA against *HDGF. HDGF* expression was analyzed 48 h after siRNA transfection by qRT-PCR. Results were normalized to si-control. ****p<0.0001; one-way ANOVA with Dunnett’s multiple comparisons test. **(B)** HDGF and ß-catenin protein expression was determined 48 h after siRNA transfection by western blotting with cofilin as loading control. Densitometric analysis of western blot assays is shown in Fig. S9F. **(C)** HLF and Huh6 cells were transfected with 10 nM siRNA. Cell viability was analyzed by WST-1 assay and normalized to si-control (dotted line). Apoptosis was analyzed by Caspase 3/7 assay and normalized to cell viability and si-control (dotted line). *p < 0.05, **p < 0.01, ***p < 0.001; two-way ANOVA with Dunnett’s multiple comparisons test. **(D)** Migration capacity of HLF cells was analyzed by transwell assay. ***p < 0.001, ****p<0.0001; one-way ANOVA with Dunnett’s multiple comparisons test; scale bar = 200.00 µm **(E)** HLF and Huh6 cells were transfected with 10 nM siRNA against *HDGF*. 48 h after transfection, protein expression of p-ERK1/2 and ERK1/2 was analyzed by western blotting with cofilin as loading control. Gels were processed in parallel. Densitometric analysis of western blot assays is shown in Fig. S9G.


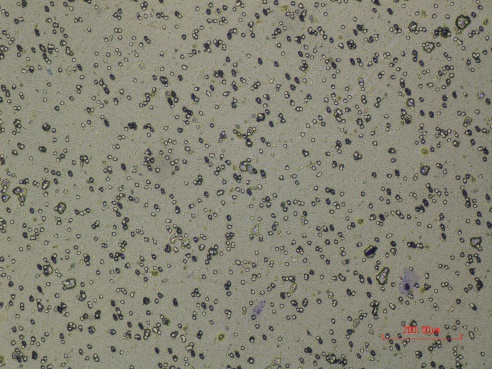

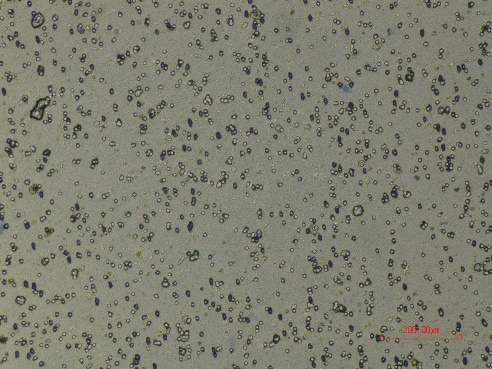

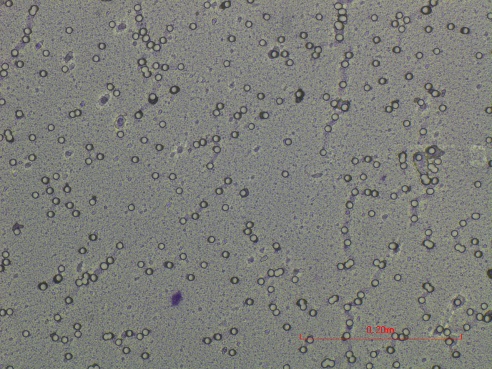

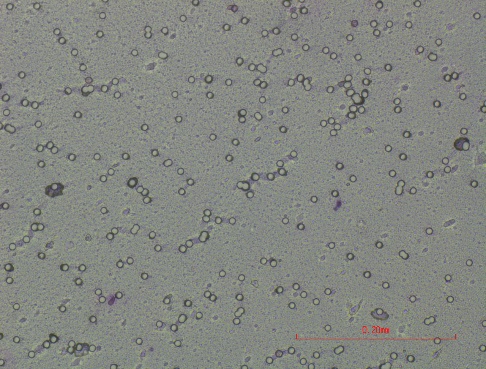

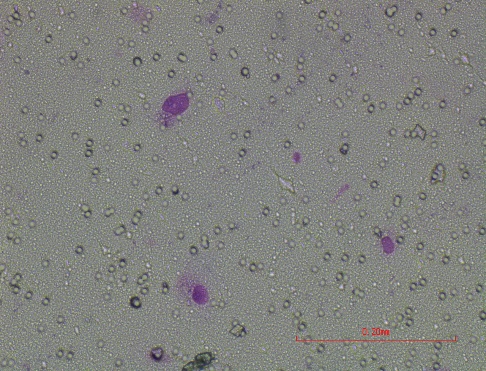

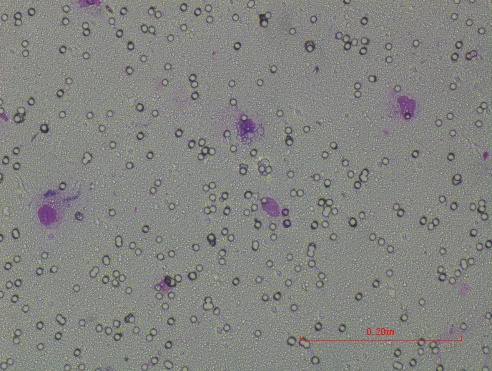


14 h

20 h

HepG2

Huh6

Huh7

Figure S8 Migration capacity of HepG2, Huh6, and Huh7 cells

Cell migration of HepG2, Huh6, and Huh7 cells was analyzed by transwell assays at different times, but no migration of HepG2 and Huh6 cells was detectable. Huh7 cells only showed a very weak migration so that no differences between miR-control and miR-129-5p-treated cells could be detected; scale bar = 200.00 µm.

#

**A** (Fig. 2E)


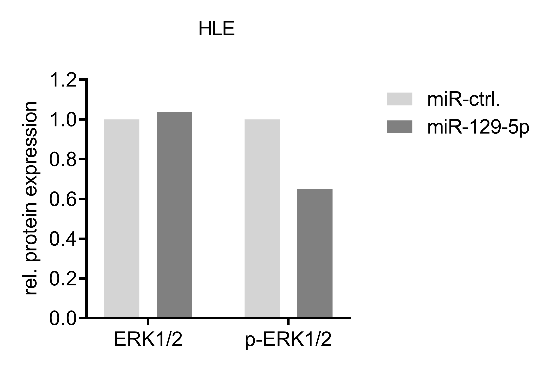

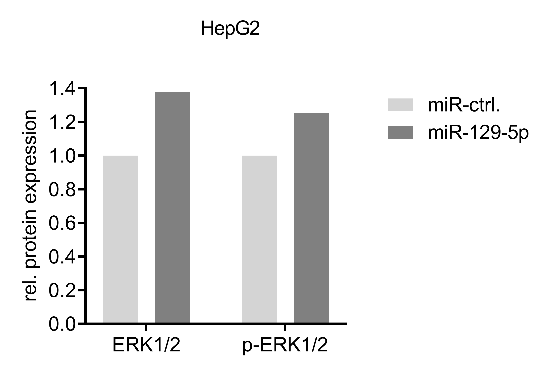

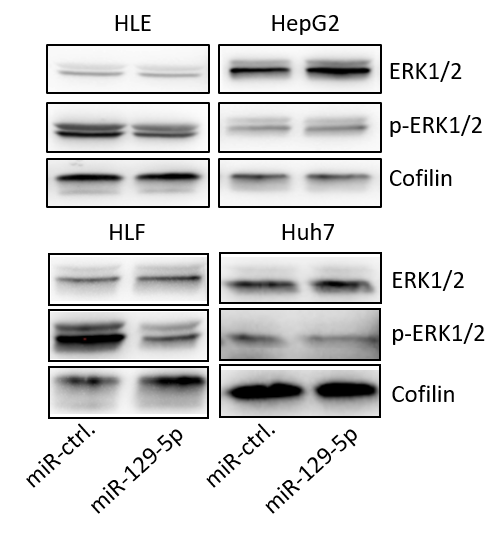

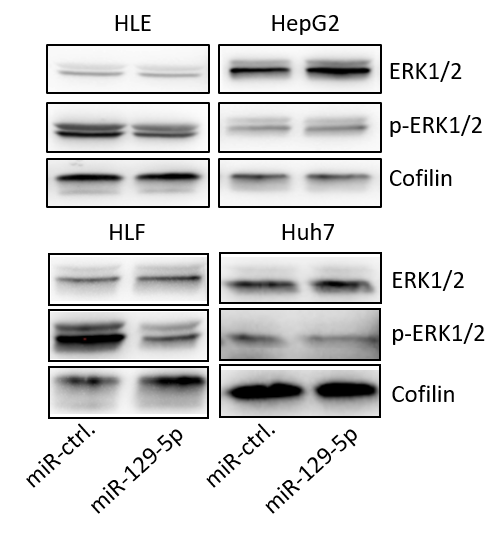

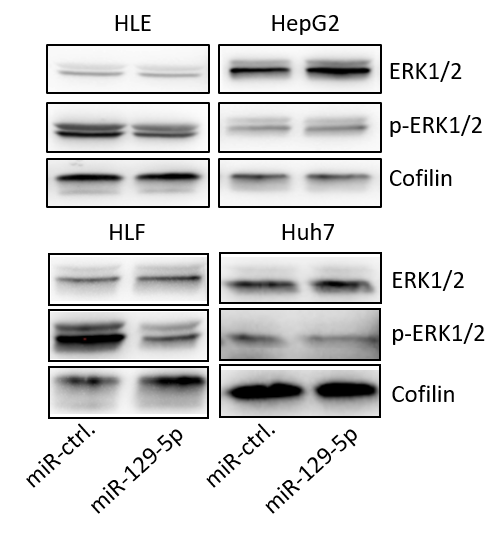

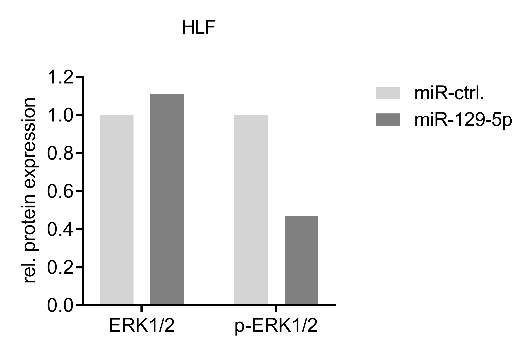

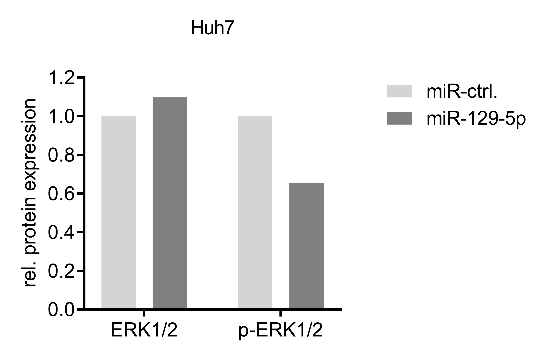


**B** (Fig. 3D)


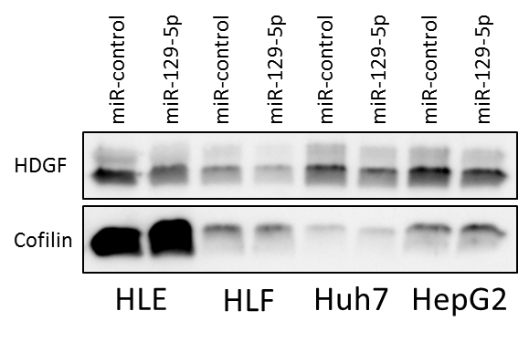

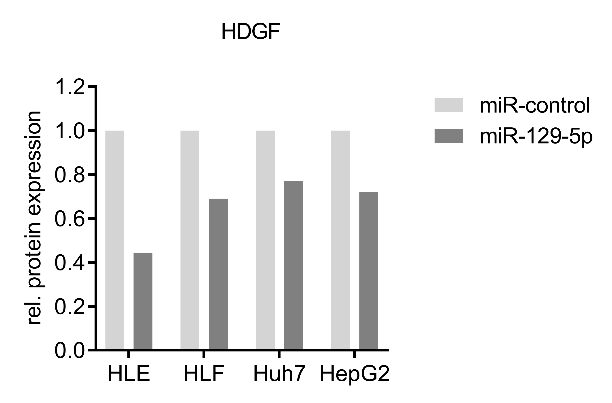


**C** (Fig. 5B)


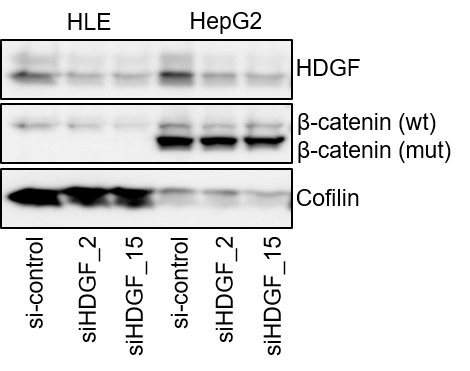

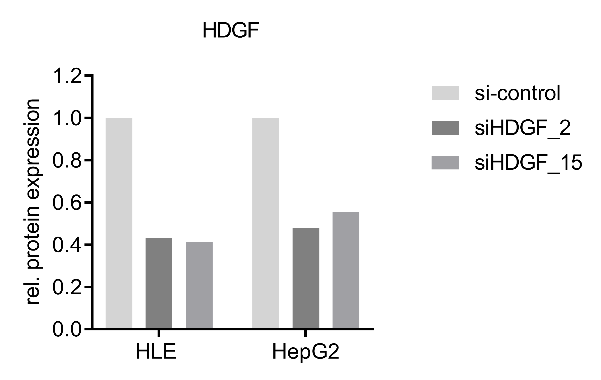

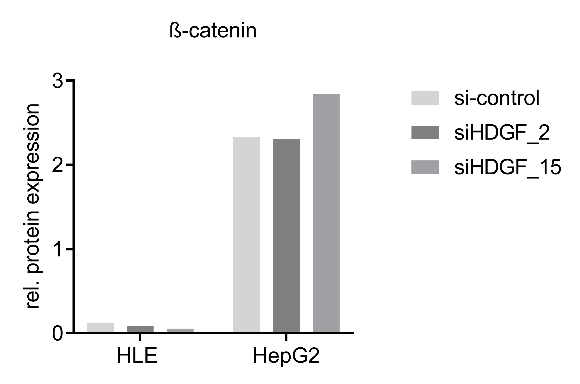


**D** (Fig. 5E)


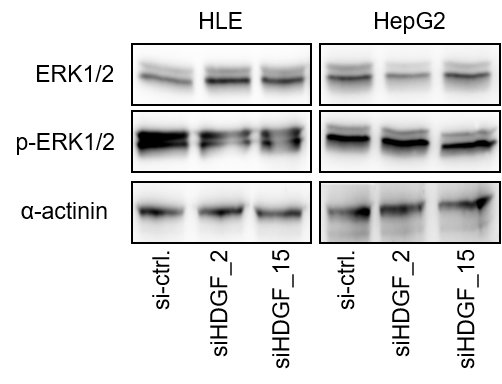

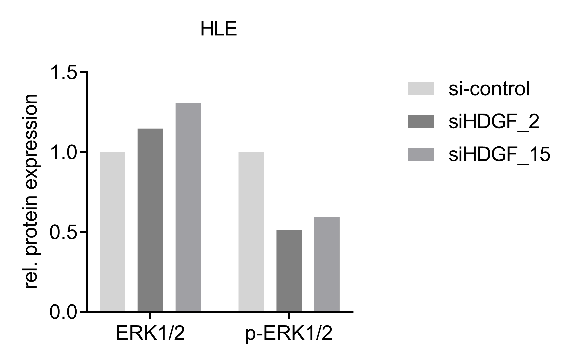

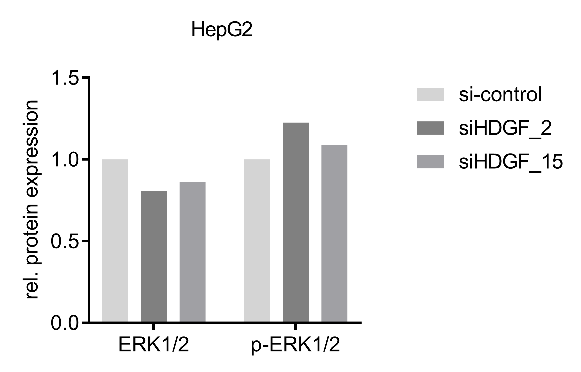


**E** (Fig. S3B)


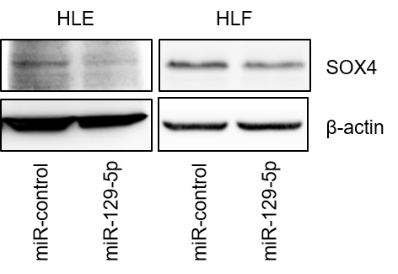

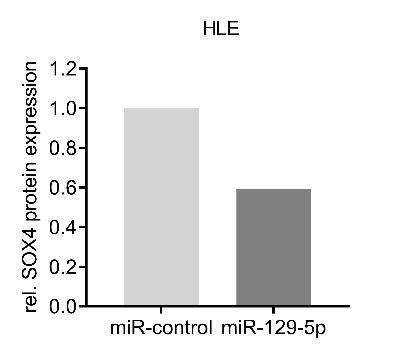

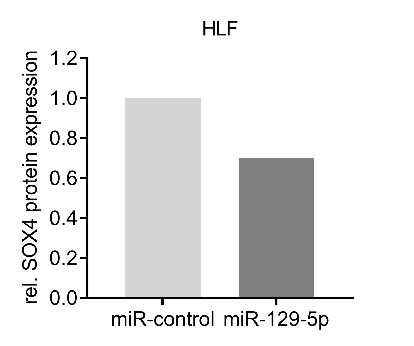


**F** (Fig. S7B)


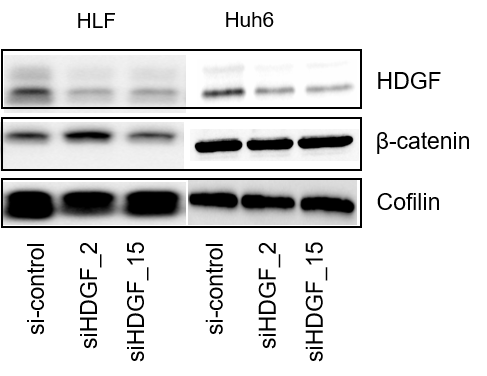

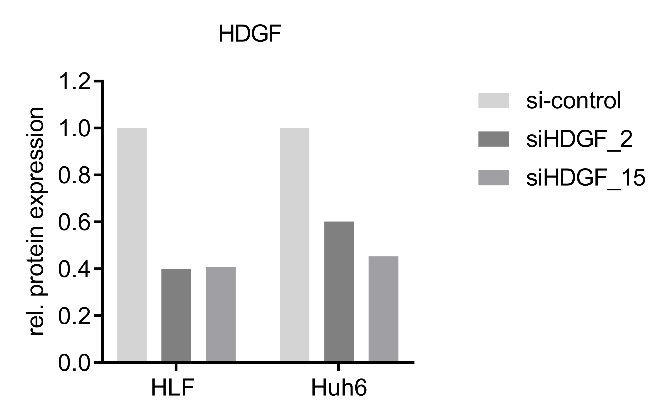

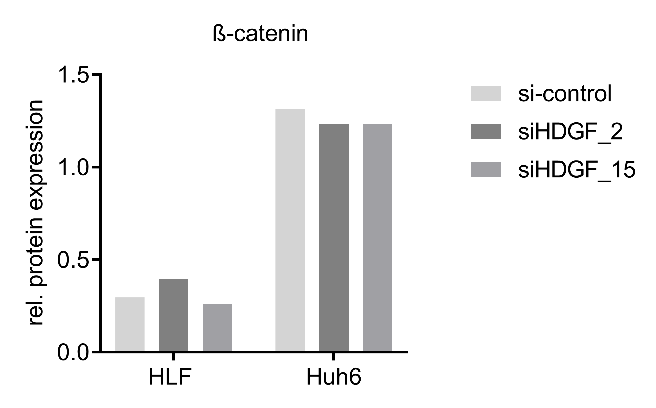


**G** (Fig. S7E)


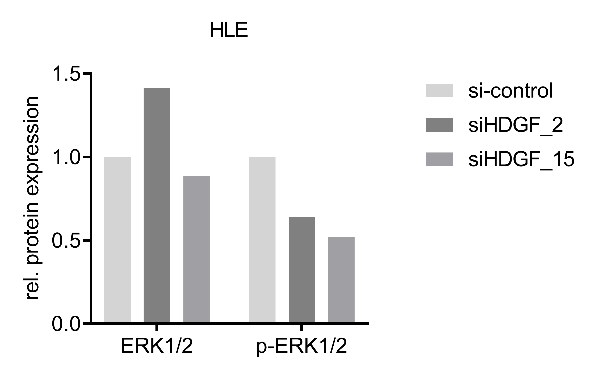

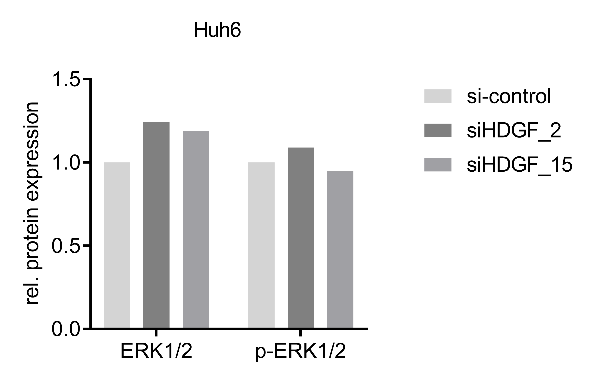

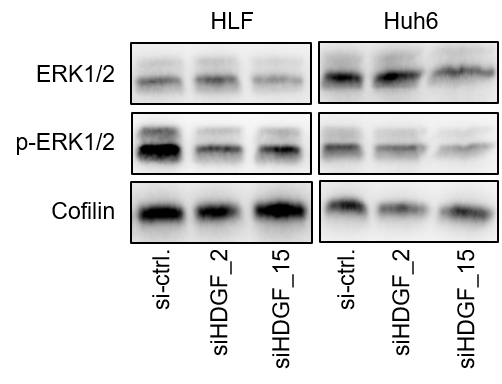


Figure S9 Densitometric analysis of western blot assays

Densitometric analysis of western blot assays was performed using Image Studio™ Software. Protein expression was normalized to loading control.

Additional Tables

Table S1 Primer for cloning of luciferase reporter vectors

| **Primer** | **Sequence (5’ – 3’)** |
| --- | --- |
| *HDGF*-3’UTR (68-1253) fw | *GATCTCTAGA*GCCATGGCCTGCAAACTG |
| *HDGF*-3’UTR (68-1253) rv | *GATCGGCCGGCC*CATCAGAGCCCAAGGGAGAG |
| *HDGF*-3’UTR (765-1253) fw | *GATCTCTAGA*GAAGCCACAAATAGGATGC |
| *HDGF*-TargetScan-mut (68-1253) fw | GTTTTTAATAAAAGAAAATTAC**GT**A**GTC**AAATTTTAAAGACCCC |
| *HDGF*-TargetScan-mut (68-1253) rv | GGGGTCTTTAAAATTT**GAC**T**AC**GTAATTTTCTTTTATTAAAAAC |

Table S2 siRNAs and miRNA mimic for transfection

| **Oligonucleotide** | **Order no.** | **Manufacturer** |
| --- | --- | --- |
| Allstars Negative Control | SI03650318 | Qiagen, Hilden, Germany |
| siHDGF_2 | SI00300398 | Qiagen, Hilden, Germany |
| siHDGF_15 | SI04351690 | Qiagen, Hilden, Germany |
| syn-has-miR-129-5p | MSY0000242 | Qiagen, Hilden, Germany |

Table S3 gBlocks gene fragments (IDT)

| **gBlock** | **Sequence (5’ – 3’)** |
| --- | --- |
| *HDGF*-3’UTR (971-1253) | *ATGATCTCTAGA*CCATCGTTGCCCCCATTTCTGAGGTGCACTGGGAGGCTCCCCTTCTATTTGGGGCTTGATGACTTTCTTTTTGTAGCTGGGGCTTTGATGTTCCTTCCAGTGTCATTTCTCATCCACATACCCTGACCTGGCCCCCTCAGTGTTGTCACCAGATCTGATTTGTAACCCACTGAGAGGACAGAGAGAAATAAGTGCCCTCTCCCACCCTCTTCCTACTGGTCTCTCTATGCCTCTCTACAGTCTCGTCTCTTTTACCCTGGCCCCTCTCCCTTGGGCTCTGATG*GGCCGGCCATGATC* |
| *HDGF*-IntaRNA1-mut (971-1253) | *ATGATCTCTAGA*CCATCGTTGCCCCCATTTCTGAGGTGCACTGGGAGGCTCCCCTTCTATTTGGG**TA**TTGAT**TCA**TTTCTTTTTGTAGCTGGGGCTTTGATGTTCCTTCCAGTGTCATTTCTCATCCACATACCCTGACCTGGCCCCCTCAGTGTTGTCACCAGATCTGATTTGTAACCCACTGAGAGGACAGAGAGAAATAAGTGCCCTCTCCCACCCTCTTCCTACTGGTCTCTCTATGCCTCTCTACAGTCTCGTCTCTTTTACCCTGGCCCCTCTCCCTTGGGCTCTGATG*GGCCGGCCATGATC* |
| *HDGF*-IntaRNA2-mut (971-1253) | *ATGATCTCTAGA*CCATCGTTGCCCCCATTTCTGAGGTGCACTGGGAGGCTCCCCTTCTATTTGGGGCTTGATGACTTTCTTTTTGTAGCTGGGGCTTTGATGTTCCTTCCAGTGTCATTTCTCATCCACATACCCTGACCTGGCCCCCTCAGTGTTGTCA**AACTC**T**A**TGATTTGTAACCCACTGAGAGGACAGAGAGAAATAAGTGCCCTCTCCCACCCTCTTCCTACTGGTCTCTCTATGCCTCTCTACAGTCTCGTCTCTTTTACCCTGGCCCCTCTCCCTTGGGCTCTGATG*GGCCGGCCATGATC* |

Table S4 TaqMan Assays for quantitative real-time PCR

| **Target gene / miRNA** | **Assay ID** | **Manufacturer** |
| --- | --- | --- |
| CDH1 | Hs01023895_m1 | Life Technologies, Carlsbad, CA, USA |
| HDGF | Hs00610314_m1 | Life Technologies, Carlsbad, CA, USA |
| pri-miR-129-1 | Hs03302824_pri | Life Technologies, Carlsbad, CA, USA |
| pri-miR-129-1 | Hs03303241_pri | Life Technologies, Carlsbad, CA, USA |
| SOX4 | Hs04987498_s1 | Life Technologies, Carlsbad, CA, USA |
| TBP | Hs00920494_m1 | Life Technologies, Carlsbad, CA, USA |
| VIM | Hs00185584_m1 | Life Technologies, Carlsbad, CA, USA |
| miR-129-5p | 590 | Life Technologies, Carlsbad, CA, USA |
| RNU6B | 1093 | Life Technologies, Carlsbad, CA, USA |

Table S5 Antibodies for AGO2-IP

| **Antigen** | **Order no.** | **Manufacturer** |
| --- | --- | --- |
| Argonaute 2 | 11A9-100 | Chromotek, Planegg-Martinsried, Germany |
| HA-tag (IgG) | 7c9-100 | Chromotek, Planegg-Martinsried, Germany |
